# Single-cell RNA-Seq resolves cellular complexity in sensory organs from the neonatal inner ear

**DOI:** 10.1038/ncomms9557

**Published:** 2015-10-15

**Authors:** Joseph C. Burns, Michael C. Kelly, Michael Hoa, Robert J. Morell, Matthew W. Kelley

**Affiliations:** 1 Laboratory of Cochlear Development, National Institute on Deafness and Other Communication Disorders, National Institutes of Health, Bethesda, Maryland 20892, USA; 2 Genomics and Computational Biology Core, National Institute on Deafness and Other Communication Disorders, National Institutes of Health, Bethesda, Maryland 20892, USA

## Abstract

In the inner ear, cochlear and vestibular sensory epithelia utilize grossly similar cell types to transduce different stimuli: sound and acceleration. Each individual sensory epithelium is composed of highly heterogeneous populations of cells based on physiological and anatomical criteria. However, limited numbers of each cell type have impeded transcriptional characterization. Here we generated transcriptomes for 301 single cells from the utricular and cochlear sensory epithelia of newborn mice to circumvent this challenge. Cluster analysis indicates distinct profiles for each of the major sensory epithelial cell types, as well as less-distinct sub-populations. Asynchrony within utricles allows reconstruction of the temporal progression of cell-type-specific differentiation and suggests possible plasticity among cells at the sensory–nonsensory boundary. Comparisons of cell types from utricles and cochleae demonstrate divergence between auditory and vestibular cells, despite a common origin. These results provide significant insights into the developmental processes that form unique inner ear cell types.

## Abstract (web summary)

Heterogeneous sensory epithelia of the inner ear are difficult to study owing to the few cells that can be isolated. Here the authors provide insight into the developmental processes underlying the formation of these cells by single-cell RNA-Seq.

## Graphical abstract

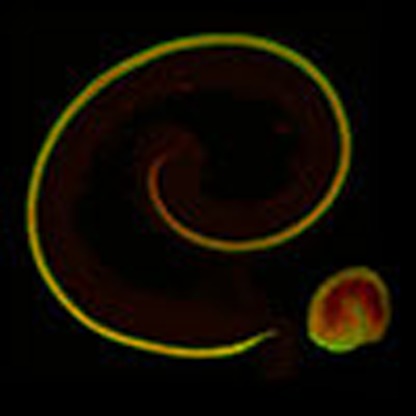

The mouse inner ear contains five vestibular sensory epithelia specialized for detection of linear and rotational acceleration and a single auditory epithelium, the organ of Corti. Each of these epithelia contains two primary cell types, hair cells (HCs) and supporting cells (SCs), arranged in exquisite mosaic patterns (Fig. 1a–g). While HCs and SCs appear grossly homogeneous, anatomical features, physiological characteristics and pharmacological sensitivity suggest the existence of unique sub-populations of both cell types in each epithelium^1^,^2^,^3^,^4^,^5^,^6^,^7^,^8^,^9^. For instance, at birth, HCs and SCs within the striola of the utricle, a crescent-shaped zone near the centre of the epithelium, which has been suggested to play a role in perception of rapid head movements, appear to differ from those in extrastriolar regions^8^,^10^,^11^, whereas in the organ of Corti, HCs and SCs are segregated into medial and lateral compartments with unique functional roles (Fig. 1a–g; Supplementary Fig. 1). Furthermore, HCs within the early-postnatal mouse utricle probably comprise a greater degree of heterochrony by comparison with their cochlear counterparts. In the cochlea, the majority of HC production is tightly synchronized and occurs during a relatively brief period between E13–E17; however, HCs in the utricle arise more sporadically over an extended period of time that spans E13–P12 (refs 12, 13, 14, 15). Finally, cells in both organs undergo further postnatal refinement and maturation with fully mature phenotypes not present until at least 2 weeks after birth. HCs differentiate into subtypes with distinct electrophysiological traits (extrastriolar and striolar type-I and type-II HCs in the utricle and inner and outer HCs in the cochlea), and SCs develop elaborate cytoskeletal structures leading to unique morphologies, which in the cochlea can be categorized into at least five subtypes: inner phalangeal cells, inner and outer pillar cells, Deiters' cells and Hensen's cells.

This intricate heterogeneity is constructed on an extremely small scale. By comparison with other sensory structures, such as the retina, the number of sensory cells within the inner ear is three orders of magnitude smaller—approximately 7 million cells in the mouse retina versus ∼6,000 HCs and SCs in the sensory regions of either the mouse cochlea or utricle^12^,^16^,^17^,^18^. As a result, characterization of transcriptional profiles for unique HC or SC sub-populations has been challenging, although RNA sequencing (RNA-Seq) of bulk populations of HCs purified mechanically or with fluorescence-activated cell sorting (FACS) has been reported^19^,^20^,^21^. Here, we show that single-cell RNA-Seq can be used to characterize transcriptome-wide heterogeneity among individual HCs and SCs isolated from the utricles and cochleae of neonatal mice. We uncover novel, molecular-level differences between HCs and SCs, and we find that intra-cell-type diversity at this stage is dominated by temporal and regional differences.

## Results

### RNA-Seq of single cells from inner ear sensory epithelia

**Figure 1:**
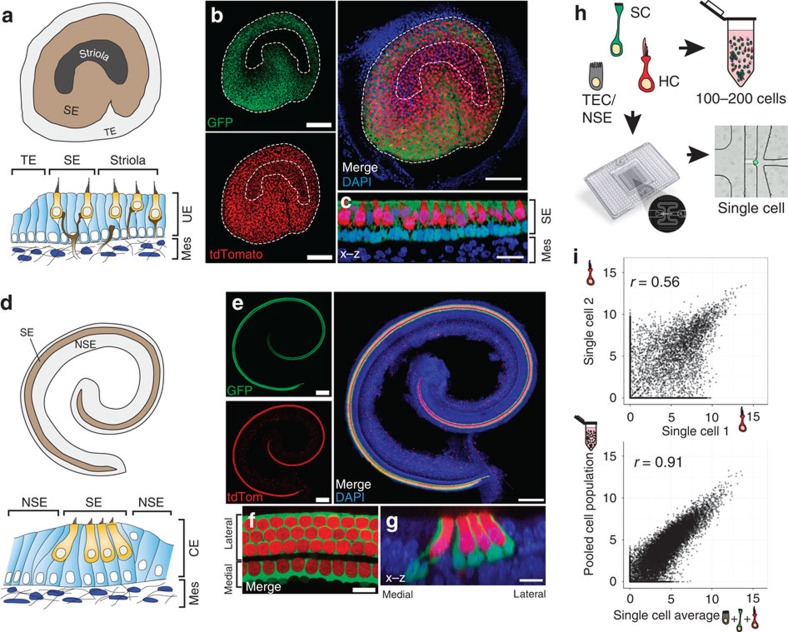
Genetic labelling and RNA-Seq of single cells from the newborn mouse inner ear. (**a**) Diagrams depicting regional heterogeneity in the utricle, a linear acceleration detector. Surface view (top) shows the sensory epithelium (SE), which contains HCs and SCs, and the surrounding transitional epithelium (TE) that is devoid of HCs and SCs. The striola is a crescent-shaped zone that sits in the centre of the SE where specialized HCs and SCs may reside. Cross-sectional view (bottom) illustrates that the utricular epithelium (UE) sits on a matrix (Mes) that contains mesenchyme and neuronal processes. (**b**,**c**) Genetic labelling of SCs and HCs in *Lfng*^*EGFP*^; *R26R*^*CAG-tdTomato*^; *Gfi1*^*Cre*^ mice at P1. In extra-striolar regions, SCs are GFP+/tdTomato−, and HCs are GFP+/tdTomato+. In contrast, GFP is expressed at or below the level of detection in most striolar cells (outlined). (**d**–**g**) Comparable images as in **a**–**c** for the cochlear epithelium. The coiled cochlea contains a narrow strip of HCs and SCs (SE) bounded on both the medial and lateral sides by non-sensory epithelium (NSE). In P1 cochleae from *Lfng*^*EGFP*^; *R26R*^*CAG-tdTomato*^; *Gfi1*^*Cre*^ mice, nearly all HCs are tdTomato+, and all SCs except inner pillar cells (see Supplementary Fig. 2 for details) are GFP+. Mesenchymal cells express tdTomato (tdTom) as well, but are excluded by epithelial delamination. (**h**) Workflow for preparing inner ear cells for RNA-Seq. Dissociated HCs, SCs and TECs/NSE from utricle or cochlea were isolated and prepared for single-cell RNA-Seq on a C1 IFC and then imaged before lysis. For comparison, some dissociated samples were prepared as 100–200-cell bulk populations. Single cells and bulk tube controls were prepared and processed in the same manner. (**i**) Correlation plots of log_2_(nTPM) gene expression for all 26,583 genes in the NCBI-annotated mouse genome for two randomly selected HCs (top) and the average of all single cells compared with a tube control (bottom). The increase in *r*-value (Spearman's correlation) when all single cells are compared with a tube control suggests that much of the variation between individual cells is biological. Scale bars, 100 μm (**b**); 20 μm (**c**); 200 μm (**e**); 10 μm (**f**,**g**).

The recent development of microfluidics-based protocols for the capture of single cells and subsequent generation of high-quality complementary DNA (cDNA) libraries provides a novel method for the identification of HC and SC subtypes, as only a few thousand isolated cells are required for capture^22^,^23^. Further, isolation and quantitative profiling of transcripts from single inner ear cells has been shown to be feasible^24^. Thus, we sought to generate RNA-Seq-based transcriptomic data for single cells derived from the P1 utricle and cochlea. To identify HCs and SCs following isolation, *Lfng*^*EGFP*^; *R26R*^*CAG-tdTomato*^; *Gfi1*^*Cre*^-triple transgenic mice were generated by crossing existing lines^25^,^26^,^27^. At P1, essentially all HCs in both the utricle and cochlea express tdTomato driven by *Gfi1*^*Cre*^, whereas <1% of SCs are tdTomato+ (Fig. 1b,c,e–g; Supplementary Figs 1 and 2). In addition, most SCs in the utricle and cochlea express high levels of green fluorescent protein (GFP), driven by the *Lfng* promoter. Many utricular HCs and some cochlear HCs also express GFP, but generally at lower levels. Striolar SCs and transitional epithelial cells (TECs) located at the border between sensory and non-sensory regions in the utricle, and inner pillar cells and non-sensory cells (NSCs) in the cochlea express low or undetectable levels of both fluorescent proteins. Sensory epithelia and some surrounding TECs or NSCs were isolated from both utricle and cochlea using a combined mechanical/enzymatic technique (Supplementary Fig. 3). After dissociation into single-cell suspensions, individual cells from either organ were captured on separate integrated fluidics circuit chips (IFCs) using an automated Fluidigm platform (Fig. 1h; Supplementary Figs 3 and 4), which subsequently performs lysis, SMARTer-based reverse transcription (RT) of polyadenylated RNA and long-distance PCR amplification of resulting cDNA, for each cell within an isolated chamber on the IFC^23^. Before lysis, quality and fluorescence of each captured cell was recorded using an automated imaging platform (Fig. 1h). On the basis of quality criteria (Methods), 158 utricular cells (captured on 4 IFCs) and 91 cochlear cells (captured on 2 IFCs) were retained for further analysis following RNA-Seq (Supplementary Table 1).

There was substantial variability in gene expression between single cells (Fig. 1i; Supplementary Fig. 4f), as reported previously^22^,^23^,^28^,^29^, but the average expression across single cells was highly correlated with expression in pooled samples of 100–200 cells. Analysis of 92 RNA standards of known quantities that were added (spiked in) to the lysis buffer also indicated that sensitivity was sufficient to reliably detect transcript levels spanning six orders of magnitude (Supplementary Fig. 4e).

### Transcriptomes of cells from the P1 utricular epithelium

**Figure 2:**
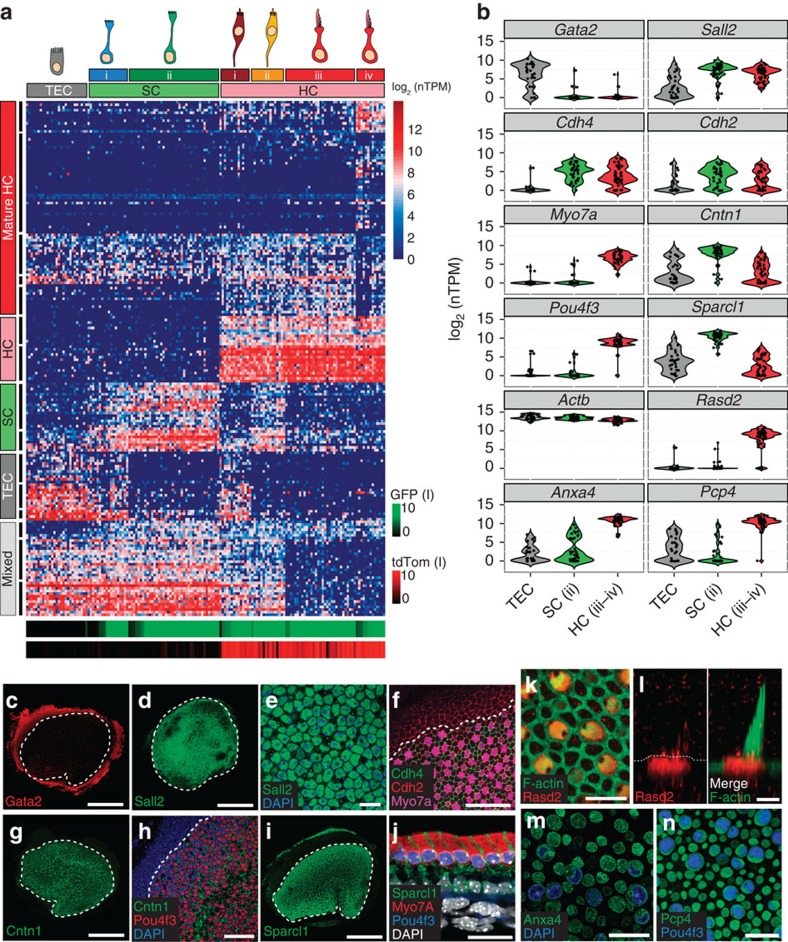
Single-cell RNA-Seq identifies unique cell types and novel markers in the newborn mouse utricle. (**a**) *K*-means clustering of 158 P1 utricle cells (*x* axis) using the top 195 genes (*y* axis) identified with PCA. The gap statistic, an unbiased estimate of the total number of clusters, identified seven distinct clusters. On the basis of expression of known marker genes and fluorescence state (bottom *x*-axis bars and right legends, displayed on log_2_ scale), each cluster was assigned to one of the three known cell types (top *x*-axis bars): TECs (grey), SCs (light green) and HCs (light red). TECs comprise one cluster, whereas SCs and HCs comprise two (SC.i–ii) and four (HC.i–iv) clusters, respectively. Genes were also divided by *k*-means (black bars indicate 15 clusters identified by gap statistic) and pooled based on cell type, HC maturity or variation across multiple cell types (Mixed). The Mixed group consists of two gene clusters expressed in non-HCs and one small cluster restricted to SCs and HCs. (**b**) Violin plots for representative genes identified as differentially expressed (FDR<0.05) between the clusters of differentiated, non-fate-transitioning cells (TEC, SC.ii and HC.iii–iv) as indicated in **a**. Black dots show the expression level for each cell. The housekeeping gene *Actb* is included for comparison. (**c**–**n**) Immunohistochemical validation of differentially expressed genes illustrated in **b**. Gata2 is enriched in TECs (**c**), whereas spalt-like transcription factor 2 (Sall2), R-cadherin (Cdh4), contactin 1 (Cntn1) and SPARC-like 1 (Sparcl1) are enriched within the sensory epithelium (SE, **d**–**j**). Co-staining of Cdh4 with N-cadherin (Cdh2) shows that Cdh4 is a more specific marker of the SE boundary (dotted line in **f**). Pou4f3 (**h**,**j**,**n**) and myosin VIIA (**f**,**j**; Myo7a) are canonical HC markers. Higher magnification or cross-sections indicate the Cntn1 (**h**) and Sparcl1 (**j**) are enriched within SCs. Rasd2, Anxa4 and Pcp4 are novel HC markers (**k**–**n**). Rasd2 localizes to the cuticular plate and base of stereocilia (**k**,**l**), Anxa4 localizes to cell membranes (**m**) and Pcp4 localizes to the cytoplasm (**n**). Scale bars, 200 μm (**c**,**d**,**g**,**i**); 10 μm (**e**,**k**); 20 μm (**f**,**j**,**m**,**n**); 50 μm (**h**); 3 μm (**l**).

**Figure 3:**
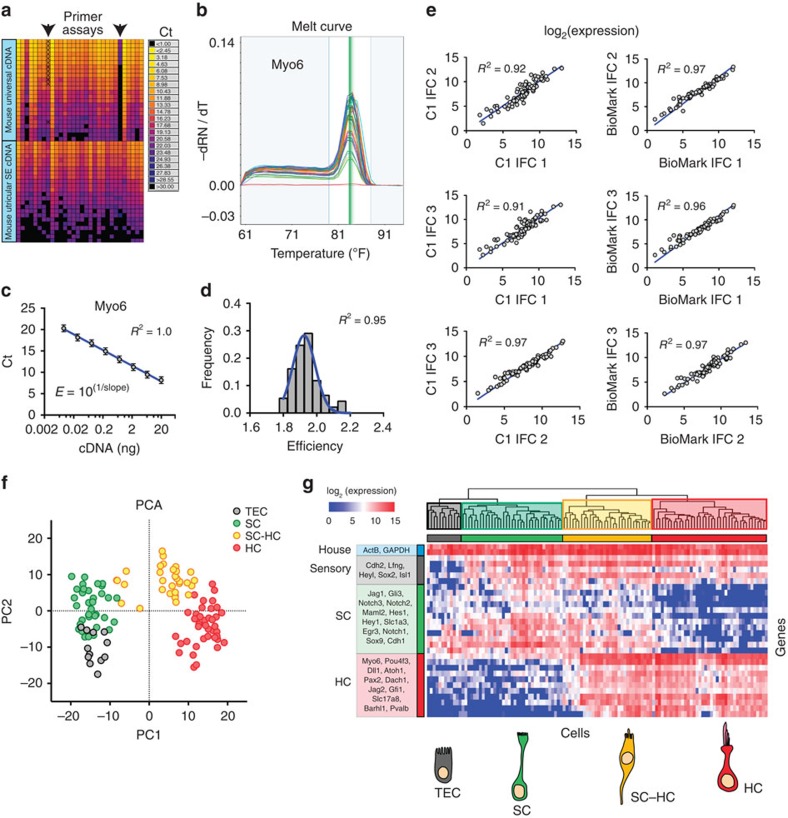
Single-cell qPCR identifies distinct cell types in the P1 utricle. (**a**) Heatmap of qPCR Cts measured from serial dilutions of mouse universal or utricular sensory epithelium (SE) cDNA, which were used to test primer efficiency. Arrows point to two assays, *Egr3* and *Pou4f3*, that showed bimodal melt curves or were not detected in universal cDNA but passed quality control metrics for utricular SE cDNA. (**b**) Example melt curves for amplicons obtained with primers for *Myo6*. Single peaks at a similar melt temperature were observed in all single cells. Assays that showed more than one peak were removed from analysis. (**c**) Plot of average Ct value measured on the BioMark system across three replicates versus input cDNA concentration (mouse universal cDNA). (**d**) Histogram of primer efficiencies for all 30 assays used. Efficiencies are normally distributed (Gaussian fit) and have an average efficiency of 1.9. (**e**) Comparison of average gene expression in single-cell qPCR data between different capture (C1) and qPCR (BioMark) IFC's. Left: graphs compare average expression of 30 genes across all cells captured on each C1 IFC for single-cell qPCR. Expression level is displayed as log_2_(expression), which is equivalent to the difference between the limit of detection Ct value and the measured Ct value. Right: graphs compare average gene expression across all cells profiled on each BioMark IFC. Cells from different C1 captures were profiled on each BioMark IFC to test for variability in the qPCR system. All plots show strong correlations (Pearson's *r*) and linear dependence, indicating that data collected on the microfluidics platforms are reproducible. (**f**,**g**) Plots show PCA (**f**) and hierarchical clustering (**g**) of 118 single cells based on qPCR-based detection of 30 genes. TECs, SCs and HCs cluster into defined groups enriched for known marker genes. A group of HCs that express SC genes (SC–HC, yellow) can also be identified. The 30 assays utilized did not provide sufficient resolution to detect the group of HCs with expression of TEC genes (HC.i), which was identified by single-cell RNA-Seq.

Next, principal component analysis (PCA) was used to identify the genes that accounted for the majority of the variance in expression between the 158 utricular cells. Unbiased clustering with the reduced gene list identified seven clusters of cells that were readily assigned to the three primary cell populations and 15 clusters of genes within five characteristic gene groups (Fig. 2a). With few exceptions, the primary cell populations correlated with fluorescence state, indicating that they represent TECs (grey boxes), SCs (green boxes) and HCs (red boxes). The gene groups that define each population also confirmed cell-type identity, as they contain many known markers—including *Myo7a*, *Pou4f3*, *Gfi1*, *Pvalb*, *Otof*, *Calb2* and *Ptprq* in HCs; *Otog*, *Otoa*, *Gjb2*, *Gjb6*, *Jag1*, *Hes1* and *Slc1a3* in SCs; *Gata2*, *Lmx1a*, *Slc26a4* and *Cldn8* in TECs (Fig. 2a; Supplementary Data 1). In addition, we used Monocle to test for genes that were differentially expressed (false discovery rate (FDR)<0.05) and identified 1,240 putative cell-type-specific genes after filtering by specificity score (specificity>0.5, see Methods; Supplementary Data 2). These genes included more known markers, as well as genes not previously known to be specific to each cell type (Supplementary Fig. 5). We used immunohistochemistry to validate a small complement of the differentially expressed genes (Fig. 2b–n). Finally, validity of TEC, SC and HC populations was confirmed by measuring expression of 30 known genes in an additional 118 P1 utricle cells using single-cell quantitative (q)PCR (Fig. 3a–g).

### Resolution of fate transitions suggests a novel source of HCs

Next, we examined the seven cell clusters that comprise the three major populations. One cluster contains all TECs, whereas SCs comprise two clusters (SC.i–ii) and HCs consist of four clusters (HC.i–iv, Fig. 2a). SC.i and HC.i–ii appear to represent transitional cell types that co-express combinations of TEC, SC and HC genes (Fig. 2a). By contrast, SC.ii and HC.iii–iv are more distinct and do not express genes characteristic of other populations. HC.iv expresses a subset of genes that are unique to this group, suggesting that these cells may be more differentiated. However, based on the limited number of these cells, we grouped HC.iii and HC.iv into a single ‘mature' population for subsequent analyses.

**Figure 4:**
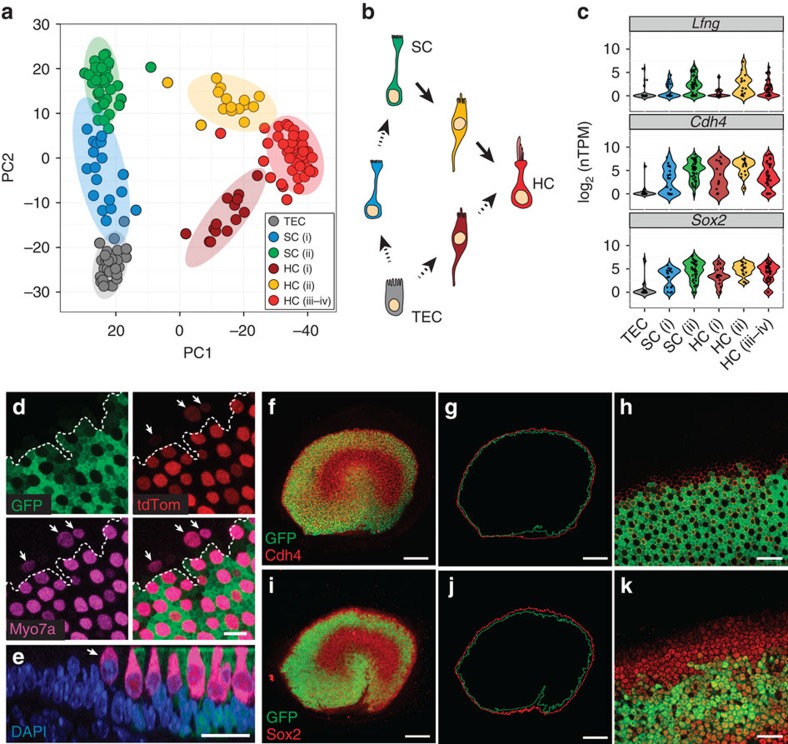
Single-cell RNA-Seq identifies cellular transitions and possible variation in the sensory/non-sensory boundary. (**a**) Plot of P1 utricular cells projected onto the first two principal components (PCs) identified by PCA using the top 195 genes (same as Fig. 2a). Each circle is a single cell while the larger ovals represent 95% confidence regions. Cells are coloured based on the groups assigned by *k*-means clustering. (**b**) Model of cellular relationships based on the data in **a**, suggesting that the sensory epithelium adds new SCs and HCs via both interstitial and appositional growth. (**c**) Violin plots showing expression of genes that potentially mark the sensory region in the P1 utricle. Expression within all of the *k*-means clusters is shown. In the HC.i group, *Lfng* expression is low compared with *Cdh4* and *Sox2*. (**d**) High-resolution confocal image of the lateral edge of the utricular sensory epithelium from a P1 *Lfng*^*EGFP*^; *R26R*^*CAG-tdTomato*^; *Gfi1*^*Cre*^ mouse. New HCs expressing low levels of both Myo7a and tdTom (arrows) are present beyond the lateral edge of the sensory epithelium defined by *Lfng*^*EGFP*^ expression (dotted line). (**e**) Cross-sectional view of the utricle in **d** illustrating a single HC (arrow) outside of the GFP expression domain. (**f**–**h**) Confocal images of Cdh4 immunolabelling in a P1 *Lfng*^*EGFP*^ mouse utricle. Traces (**g**) around the borders of the GFP+ (green) and Cdh4+ (red) domains reveal that the Cdh4+ region is slightly larger. Single confocal z-plane (**h**) at the apical surface of the lateral edge in **f**. At most locations, two to three Cdh4+ cells extend past the last GFP+ cell. (**i**–**k**) Similar analysis as **f**–**h** for GFP and Sox2. Compared with Cdh4, the Sox2+ domain extends even further past the edge of GFP+ cells, in some instances up to five cell widths. Image in **k** is a single confocal *z*-plane at the level of the SC nuclei. Scale bars, 10 μm (**d**); 20 μm (**e**,**h**,**k**); 100 μm (**f**,**g**,**i**,**j**).

To further examine the relationships between clusters, all cells were projected onto the first two principal components (PC1 and PC2) identified from PCA (Fig. 4a). The resulting two-dimensional plot indicates clear separations of the six clusters: TECs, SC.i, SC.ii, HC.i, HC.ii and HC.iii–iv, with HCs separating from both SCs and TECs along PC1. In contrast, PC2 separates SC.ii and TECs, with SC.i falling in the middle. HC.i and HC.ii appear to be in a transitional state between SC.ii and TEC, respectively. These results suggest that SCs and HCs within the utricle may arise from two distinct precursors, SC-like progenitors within the sensory epithelium and TEC-like progenitors located outside the sensory epithelium (Fig. 4b). While the development of HCs from SCs within the sensory epithelium was known, a potential TEC–HC lineage was surprising. An alternative explanation would be that the boundary between the sensory and non-sensory regions might be more fluid than previously thought, consisting of a transitional zone where cells can readily switch fates. This sort of plasticity would be consistent with recent findings indicating that cells within the cochlear greater epithelial ridge (GER) can develop as HCs in neonates^30^,^31^.

To explore this idea further, we examined the expression patterns of putative markers of the sensory region at its lateral edge, as substantial postnatal HC production occurs at this location^12^. Lfng—as assessed by GFP expression in *Lfng*^*EGFP*^ mouse utricles—Cdh4, and Sox2 were all confined to a domain that ended at or near the outermost HCs (Fig. 4c–k). In support of a TEC–HC lineage, high-magnification images showed that some Myo7a+ cells were developing just outside the Lfng domain (Fig. 4d,e). However, we also found that the Cdh4 domain spanned several cell widths past the Lfng border, and the Sox2 domain extended even farther (Fig. 4f–k). Consistent with these observations, HC.i, the cells identified as transitioning between TECs and HCs by PCA, expressed low levels of *Lfng* but high levels of *Cdh4* and *Sox2* (Fig. 4c). This evidence supports the hypothesis that the sensory and non-sensory domains of the utricle are separated by a transitional region rather than a strict boundary.

### Temporal ordering of cells reveals kinetics of expression

**Figure 5:**
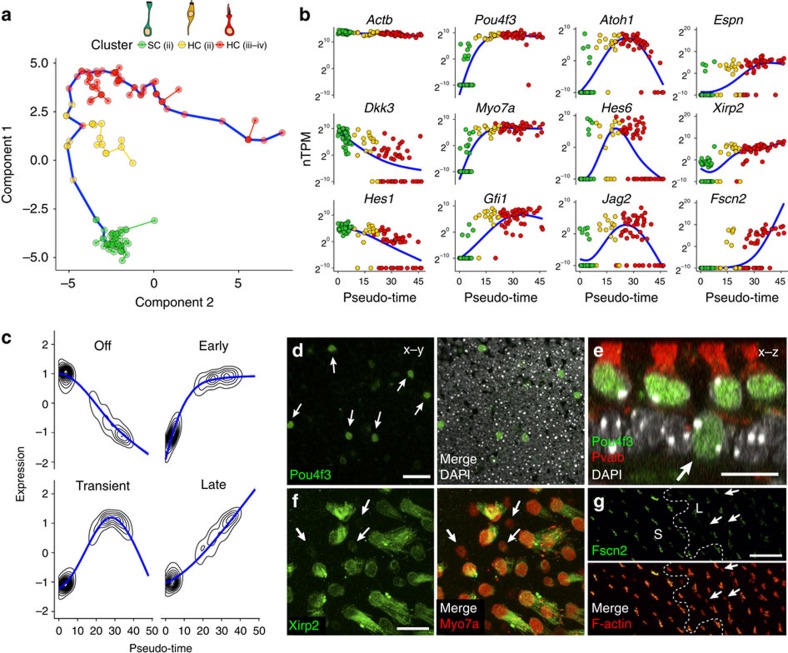
Ordering single cells along a differentiation trajectory reveals mechanistic insights into utricular HC differentiation. (**a**) Ordering of SC to HC differentiation using Monocle. On the basis of the model illustrated in Fig. 4b, cells from the SC.ii and HC.ii–iv clusters were used for ordering. Individual cells are connected by a minimum spanning tree (thin lines), and the longest line through the tree (blue) represents the differentiation trajectory (pseudo-time). (**b**) Gene expression levels in single cells ordered along the pseudo-time axis from **a**. Housekeeping genes such as *Actb* change little while SC-specific genes such as *Dkk3* and *Hes1* gradually turn off. Also shown are examples of genes expressed early (*Pou4f3*, *Myo7a* and *Gfi1*), transiently (*Atoh1*, *Hes6* and *Jag2*) or late (*Espn*, *Xirp2* and *Fscn2*) in the HC differentiation process. (**c**) Plots of average expression profiles for genes that show significant variation over pseudo-time. Genes were clustered into four groups before generating the average profiles (see Methods for details). The groups reflect the different trends observed in **c**. Topographic lines show where the segments of profiles for individual genes are concentrated along the average trend. (**d**) Single confocal *z*-plane through the SC nuclear layer near the lateral edge in a P1 mouse utricle. Several of the SC nuclei (arrows) label with an antibody to Pou4f3 (green, early gene in **b**). (**e**) Confocal cross-section through a P1 mouse utricle. Arrow points to a Pou4f3+ SC nucleus. Note that it is located in the SC nuclear layer, suggesting that it is a HC in the early stages of differentiation. All HC soma are counterstained with an antibody to Pvalb (red). (**f**,**g**) Confocal images of stereocilia labelled with antibodies to the ‘Late' HC differentiation genes, *Xirp2* (**f**) and *Fscn2* (**g**). Arrows point to small, immature-appearing HCs with little to no Xirp2/Fscn2 antibody labelling. Cuticular plates and stereocilia of larger, more mature-appearing HCs label intensely. Images were taken near the lateral edge. Dashed line in **g** indicates the line of polarity reversal. S, striola, L, lateral. Scale bars, 20 μm (**d**,**g**); 10 μm (**e**,**f**).

One of the advantages of single-cell transcriptional profiling, particularly in an organ with cells at different stages of maturity such as the P1 utricle, is that it provides an opportunity to order cells along a hypothetical timeline of development (pseudo-time). Using the existing utricular cell data, the Monocle single-cell analysis toolset^32^ was applied to order cells differentiating along the path of SC.ii to HC.iii–iv (Fig. 5a). The resulting, unbiased trajectory ordered cells along the expected path, beginning at SC.ii, passing through HC.ii and ending at HC.iii–iv. Expression of genes with known temporal patterns were plotted along pseudo-time (Fig. 5b). Genes enriched in SCs such as *Dkk3* and *Hes1* decreased over pseudo-time, while genes such as *Pou4f3*, *Myo7a*, *Gfi1*, *Atoh1*, *Hes6* and *Jag2*, which are upregulated in developing HCs^33^,^34^,^35^,^36^,^37^,^38^,^39^,^40^, increased at an early point along the trajectory (Fig. 5b). Finally, genes involved in assembly of mature stereocilia bundles, which occurs relatively late in the HC-differentiation process, including *Espn*^41^, *Xirp2* (refs 42, 43) and *Fscn2* (ref. 44), show a delay in onset relative to the other HC genes. Next, Monocle was used to define four clusters of genes with distinct kinetic trends over pseudo-time (Fig. 5c; Supplementary Data 2). The average trends show that expression of many early HC genes occurs in an abrupt, switch-like pattern at the onset of differentiation (Early), whereas silencing of SC genes occurs more gradually (Off, Fig. 5c). Monocle also identified groups of genes, such as *Atoh1* (refs 33, 37), *Hes6* (ref. 36) and *Jag2* (ref. 35) (Fig. 5b,c), that turn on early but are not sustained (Transient), and groups of genes that turn on later but continue to be expressed (Late).

To validate some of these patterns at the protein level, we performed immunolabelling for Pou4f3, Xirp2 and Fscn2 in P1 utricles. Monocle indicated that Pou4f3 has a particularly early onset, and we found Pou4f3+ nuclei residing within the SC nuclear layer (Fig. 5d,e). These cells were Myo7a−, suggesting that they were in the very earliest stages of differentiation. At the other end of the temporal spectrum, Xirp2 and Fscn2 only labelled large stereocilia bundles from mature-looking HCs (Fig. 5f,g). Smaller, more immature-appearing HCs that did not express Xirp2 and Fscn2 in their bundles were concentrated in the less developed lateral region of the utricle (Fig. 5f,g).

Finally, analysis of enriched transcription factor-binding motifs within the four gene groups provides a powerful opportunity to identify master regulatory transcription factors associated with distinct phases of differentiation. For example, iRegulon^45^ identified the Rfx transcription factor family as the top candidates associated with the group of ‘Early' genes. An accompanying study by Elkon *et al.*^46^ also identifies Rfx transcription factors from RNA-Seq of FACS-purified HCs and shows that Rfx1/3 are necessary for HC survival. On the basis of these results, cellular asynchrony at a single developmental age can be used to reconstruct multiple differentiation trajectories and identify groups of genes potentially involved in unique phases of HC differentiation.

### The striola is a distinct region in the P1 mouse utricle

**Figure 6:**
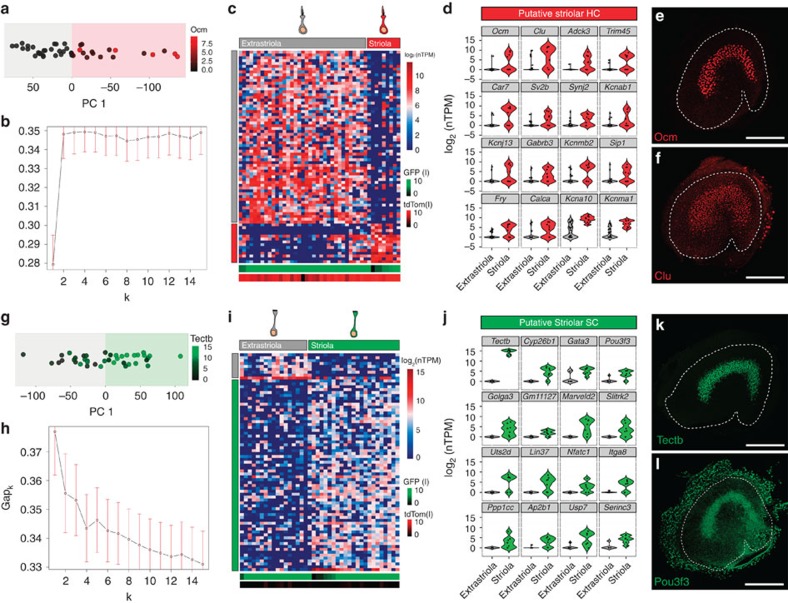
Examination of HC and SC diversity identifies the striola as a distinct region in P1 utricle. (**a**) PCA plot of the 44 cells in HC.iii–iv projected onto PC1 (all expressed genes used for PCA). Expression levels of *Ocm* in log_2_(nTPM) are indicated by the black-to-red colour gradient. (**b**) Plot of the gap statistics from *k*-means clustering with 1 to 15 clusters. The gap statistic becomes stable at two clusters. Error bars, s.e.m. (**c**) Heatmap shows *k*-means clustering of the 44 HC.iii–iv cells identified in the analyses presented in Fig. 2. The top 75 genes identified with PCA (maximum absolute eigenvector values on PC1) were used for clustering. HC.i–ii, which represent transitional cell types, were excluded from the analysis to avoid contamination from SC and TEC genes. The number of clusters was determined to be two from the gap statistic calculation in **b**. (**d**) Violin plots of representative, significant genes (FDR<0.05) found by differential expression analysis between clusters containing putative extrastriolar HCs and striolar HCs. (**e**,**f**) Immunohistochemistry for striolar HC markers Ocm (known) and Clu (novel). (**g**) PCA plot of 40 SC.ii projected onto PC1 (all expressed genes used for PCA). Expression levels of *Tectb* in log_2_(nTPM) are indicated by the black-to-green colour gradient. (**h**) Plot of gap statistics shows no clusters are identifiable in SC.ii with this metric. (**i**) Heatmap shows *k*-means clustering of differentiated SC.ii identified in the analyses presented in Fig. 2. The top 75 genes identified with PCA (maximum absolute eigenvector values on PC1) were used for clustering. SC.i, which represented transitional cell types, were excluded from the analysis to avoid contamination from TEC genes. (**j**) Violin plots of representative genes that are significantly differentially expressed (FDR<0.05) between *Tectb+* and *Tectb−* cells. (**k**,**l**) Immunohistochemistry of known (Tectb) and novel (Pou3f3) markers of striolar SCs. Scale bars, 200 μm (**e**,**f**,**k**,**l**).

One of the strengths of single-cell RNA-seq is the potential to identify novel sub-populations of cells. Therefore, we examined heterogeneity within the clusters of more mature utricular HCs and SCs. PCA using just the 44 HC.iii–iv cells revealed that PC1 separates HCs that expressed high levels of the calcium-binding protein oncomodulin (Ocm, Fig. 6a), which is specifically expressed within striolar HCs (Fig. 6e; Supplementary Fig. 1a). These putative striolar HCs clustered together (*k*-means, *k*=2, Fig. 6b,c), and subsequent differential expression testing identified 99 genes that were highly enriched within this group (FDR<0.05 and specificity>0.6; see representative examples in Fig. 6d; Supplementary Data 2), including *Ocm* (*q*=9.36 × 10^−9^). Clusterin (*Clu*), a dimeric acidic glycoprotein involved in a variety of functions including membrane recycling and apoptosis protection but not previously reported to be expressed in HCs^47^, was also differentially expressed (*q*=0.022, Fig. 6d), and antibodies to clusterin labelled a broad swath of HCs centred on the striola (Fig. 6f). These results suggest that striolar versus extrastriolar differences dominate the heterogeneity among utricular HCs at P1. This is consistent with physiological data suggesting that type-I and type-II HCs do not fully emerge until after P1 (ref. 48).

Compared with HCs, utricular SCs are thought to be relatively homogeneous^49^. However, striolar SCs specifically express the otolithic membrane glycoprotein, beta-tectorin (*Tectb*, Fig. 6k), the transcription factor Gata3 and the retinoic acid-inactivating protein, Cyp26b1 (refs 50, 51, 52). In addition, P1 striolar SCs show an enhanced propensity to regenerate lost HCs and convert into HCs after pharmacological Notch inhibition^8^,^11^, suggesting that striolar SCs are a subtype. Although PC1 clearly separated cells within SC.ii, which were expressing high levels of *Tectb* (Fig. 6g), the gap statistic failed to identify distinct clusters of cells (Fig. 6h). Since PCA suggested that the majority of the variance arose from extrastriolar versus striolar differences, we performed *k*-means with two clusters and found that one of the clusters expressed significantly higher levels of *Tectb* (*q*=0.047, Fig. 6i). However, some SCs in the contrasting cluster also expressed *Tectb*, consistent with the results from the gap statistic. To more reliably assess regional variation, we therefore tested for differential expression between *Tectb*+ and *Tectb*− cells (see Methods), and found 202 genes that were enriched within *Tectb+* SCs (FDR<0.05 and specificity>0.6; see representative striolar genes in Fig. 6j; Supplementary Data 2). The list of significant genes included *Gata3* (*q*=0.00084, Fig. 6j), *Cyp26b1* (*q*=7.94 × 10^−26^, Fig. 6j) and the transcription factor *Pou3f3* (*q*=0.00013; Fig. 6j), which had previously been localized to an undetermined utricular subdomain^53^. Antibody labelling of whole mounts confirmed that Pou3f3+ SCs are restricted to the striolar region of the sensory epithelium (Fig. 6l). Therefore, although SCs do appear to be more homogenous than HCs at P1, the combined data indicate that the striola is a molecularly distinct region within the utricle.

### Transcriptomes of cells from the P1 cochlear epithelium

**Figure 7:**
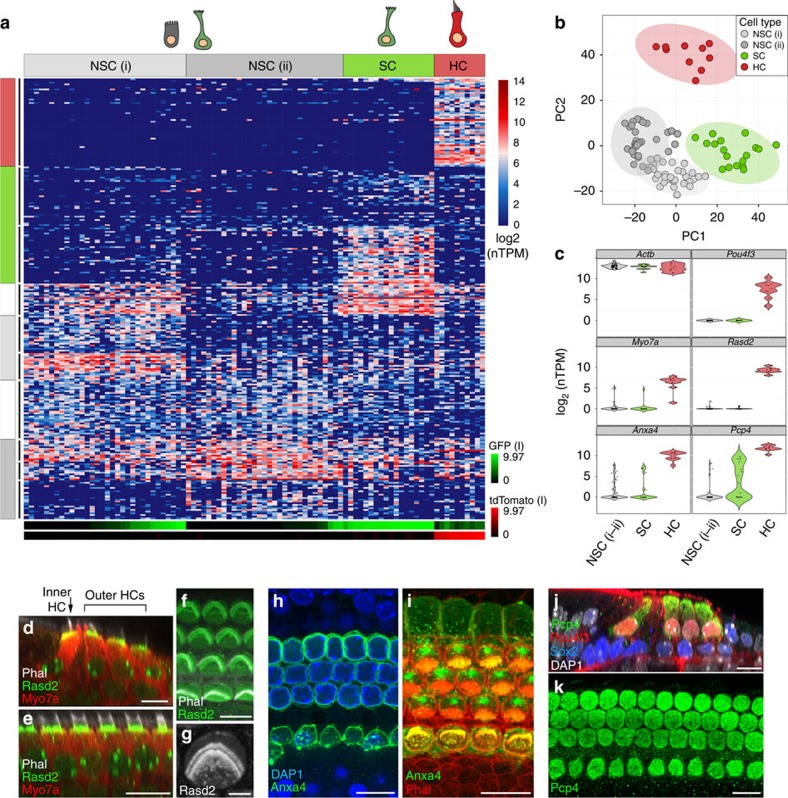
Single-cell RNA-Seq of cochlear cells reveals broad domains and identifies novel markers of auditory hair cells. (**a**) Heatmap representing the *k*-means clustering of 91 P1 cochlear epithelial cells (*x* axis) using the top 260 genes (*y* axis) identified by PCA. Gap statistics identified four cell groups, which based on known marker genes and recorded log2-transformed normalized fluorescent intensities (bottom, *x*-axis bars), were classified into known cell types (top, *x*-axis bars): NSC (grey), SC (green) and HC (red). Genes were separated into 10 *k*-means groups (left, *y*-axis bars) as specified by gap statistics, and were grouped and colour-coded by their associated enrichment with specific cell groups. Two separate *k*-group clusters (NSC.i–ii) were identified by less unique expression of gene clusters, including genes known to be differentially expressed in the medial and lateral non-sensory domains of the cochlea. Some cells within the NSC groups had high GFP expression, suggesting they are either from a subdomain of the organ of Corti, or non-sensory cells on the border (see Supplementary Fig. 2). (**b**) PCA plot of the samples and genes used in **a** across the first two principle components, depicted with the same cell group colours. (**c**) Violin plots summarizing the distribution of expression of the housekeeping gene *Actb*, the HC-specific genes *Pou4f3*, *Myo7a*, *Pcp4* and two novel genes identified and shown to be specifically expressed in HCs, *Rasd2* and *Anxa4*. (**d**–**k**) Antibody labelling of HC-specific proteins. Rasd2, localized to the base of stereocilia bundles and cuticular plates, as seen in axial (**d**), tranverse (**e**) and wholemount (**f**,**g**) views. Anxa4 (**h**,**i**) and Pcp4 (**j**,**k**) localized to HC membranes and soma, respectively. Scale bars, 10 μm (**d**–**f**,**h**–**k**); 3 μm (**g**).

As in the utricle, the organ of Corti contains both HCs and SCs but is unique in its striking arrangement of four rows of HCs and at least seven rows of SCs extending the length of the cochlear spiral (Fig. 1d–g). Moreover, cell phenotypes differ across the mediolateral axis^4^. Analysis of the transcriptional profiles of the 91 cochlear single cells following PCA-based reduction of their expressed genes reveals four major clusters of cells, which vary in their expression of distinct gene sets (Fig. 7a; Supplementary Data 1). A cluster of 10 tdTomato+ cells indicates a group of uniquely expressed genes, including the known HC genes *Pou4f3*, *Pvalb*, *Bdnf*, *Otof* and *Myo7a* (Fig. 7a, HC red boxes; Supplementary Fig. 6). In comparison, a cluster of 18 GFP+ cells express a set of genes that includes known SC genes *Lfng*, *Wnt7a*, *Fgfr3*, *Prox1* and *Hey2* (Fig. 7a, SC green boxes; Supplementary Fig. 6). Remaining cells separate into two clusters and are composed of negative cells and a limited number of GFP+ cells that expressed less clearly defined gene sets (NSC.i, NSC.ii grey boxes). Enrichment of genes known to be expressed within the region known as Kölliker's organ, such as *Gjb6*, *Cdh2*, *Lgr5* and *Fgf10* suggests that cells within NSC.i are from the medial non-sensory domain. The presence of a small number of GFP+ cells within NSC.i and the apparent enrichment for genes known to be expressed in lateral SCs within the SC cluster suggests that the GFP+ cells within NSC.i are most likely SCs from the medial domain of the organ of Corti that share gene expression with medial NSCs (Fig. 7a, light-grey boxes, *y* axis). Differential expression testing between the HC, SC and NSC.i–ii groups identified 770 putative cell-type-specific genes (FDR<0.05 and specificity>0.5, Supplementary Data 2). Of the genes differentially expressed in HCs, 30 were present in a list of 34 genes that were recently validated to be HC-specific using *in situ* hybridization^19^. Reminiscent of cells at the border of the sensory epithelium and TEC domains within the utricle, cells at the medial edge of the organ of Corti display features of active cell fate decisions, particularly at the very apical turn, which may account for some of the observed transcriptional ambiguity (Supplementary Fig. 2a–c). A two-dimensional PCA plot using the same genes and *k*-means cell group designations further shows that while the expression profiles of HCs robustly separate them from all other cochlear cell types, cells within the NSC.i cluster, in particular, have fewer expression differences that separate them from SCs (Fig. 7b). While heterogeneity within cell clusters suggests possible additional subdivisions in a tissue known to vary along spatial and differentiation gradients, the current sample size limited the confidence of such classifications, highlighting the priority of power over sequencing depth at single-cell resolution. However, unbiased clustering and conservative determination of each group based on known markers did lead to identification of novel differentially expressed genes with high confidence (Fig. 7c; Supplementary Fig. 6). We further validated three of these genes, *Rasd2*, *Anxa4* and *Pcp4*, as specifically expressed within HCs by immunolocalization (Fig. 7d–k). The protein products of these genes localized to similar regions as they did in utricular HCs (Fig. 2k–n).

### Cochlear SCs segregate along the mediolateral axis

**Figure 8:**
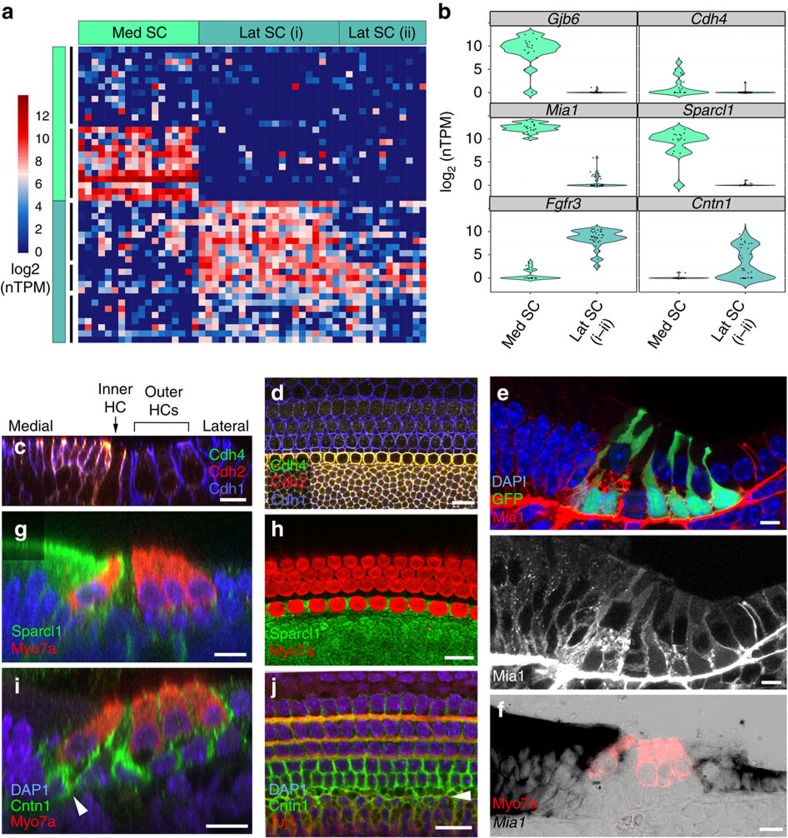
Analysis of GFP+ organ of Corti supporting cells reveals distinct medial and lateral domains. (**a**) Heatmap representing the *k*-means clustering for 52 FACS-enriched P1 GFP+ organ of Corti SCs (*x* axis) using the top 50 genes (*y* axis) identified by PCA. Gap statistics identified three sample groups (top, *x*-axis bars) and five gene groups (left, *y*-axis bars), which were labelled and colour-coded according to the expression of known markers within medial (Med) SC (teal) and lateral (Lat) SC (turquoise). Two Lat SC groups (i–ii) were identified based on varying expression of the same gene clusters. (**b**) Violin plots summarizing the distribution of expression of both known and novel genes across the two major *k*-groups (Med SC and Lat SC (i–ii)). (**c**,**d**) Antibody labelling of Cdh4 protein, showing specific expression within the cell membrane of cells within the medial domain. (**e**,**f**) Antibody (**e**) labelling and *in situ* hybridization with Myo7a antibody labelling overlay (**f**) of Mia1/*Mia1*, which shows expression within the medial sensory domain and surrounding non-sensory domains. (**g**,**h**) Sparcl1 also shows a distinct medial domain expression pattern. (**i**,**j**) Antibody labelling of Cntn1, which shows labelling in lateral domain SCs consistent with **b**, as well as in the spiral ganglion neurites (**i**, arrowhead indicating inner HC innervation), which by comparison with SCs, co-label with Tuj1 (**j**, arrowhead). Shown are orthogonal (**c**,**e**,**g**,**i**,**f**) and whole-mount (**d**,**h**,**j**) views. Scale bars, 10 μm (**c**–**j**).

By comparison with HCs, the SCs within the organ of Corti represent a relatively diverse population of cells with at least five distinct phenotypes. Using the GFP expression present within all cochlear SCs, except inner pillar cells (Fig. 1e–g), we enriched for cochlear SCs by FACS and analysed the transcriptional profiles of 53 captured GFP+ cells (Supplementary Fig. 3). Initial correlation analysis identified and allowed removal of a presumably immature GFP+ HC, which would have contaminated the averaged expression profiles of traditional population-based analysis methods (Supplementary Fig. 4). Clustering analysis of the remaining 52 GFP-enriched cells identified two main groups of cells, which express distinct gene sets (Fig. 8a). A cluster of 18 cells expresses genes that include those known to be expressed in medial SCs, including *Cdh2*, *Fabp7*, *Gjb2* and *Gjb6* (Fig. 8a, Med SC, teal boxes). The remaining 34 cells clustered into two groups, separated by variations in the levels of the same gene sets, which include genes known to be expressed in lateral SCs, such as *Cdh1*, *Prox1* and *Fgfr3* (Fig. 8a, LatSC.i–ii, turquoise boxes). In addition to previously reported genes that were differentially expressed across these two cell groups, novel markers, such as *Cdh4* (medial), *Mia1* (medial) and *Cntn1* (lateral), were identified and confirmed by antibody labelling or *in situ* hybridization (Fig. 8b–j; Supplementary Fig. 7).

### Organ-level differences between complementary cell types

**Figure 9:**
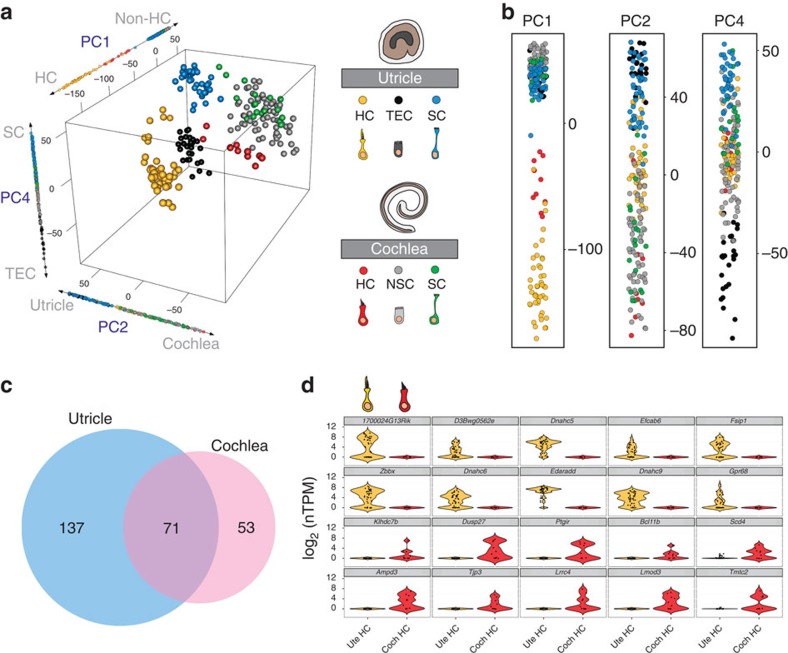
Inter-organ comparisons of single cells reveal differences between complementary cell types. (**a**) Three-dimensional PCA plot of utricular and cochlear cells (only differentiated, non-fate-transitioning utricular cells were included) onto PC1, PC2 and PC4. PC3 segregated similar cells as PC4, but to a lesser extent. See Supplementary Movie 1 for alternative angles of the three-dimensional plot. (**b**) Projections of cells onto each PC. HCs segregate from all other cells along PC1 while PC2 segregates utricular cells from cochlear cells. PC4 indicates that utricular SCs project closer to cochlear NSCs and SCs than to TECs. (**c**) Differential expression analysis across cell groups from each organ reveals genes that are shared or highly enriched within each organ. The Venn diagram shows genes that are expressed in >10 cells from one organ but no cells from the other (for all genes, FDR<0.05 and specificity score=1). See Methods for details on calculation of specificity score. The 71 shared genes were genes that failed to reach significance and found to be expressed in all cells from both organs. (**d**) Comparison of differential gene expression for cochlear (red) and utricular (yellow) HCs. Violin plots show highly specific genes enriched in each cell type (specificity score=1 for all genes).

Finally, to examine the diversity between vestibular and auditory HCs and SCs, we compared cells derived from the two organs. PCA with 14,876 expressed genes shows meaningful separation of the cells along PC1, PC2 and PC4 (Fig. 9a,b). Along these axes, each cell type separates independently with little to no mixing of cells between organs (Fig. 9a,b). PC1 separates utricular and cochlear HCs from all other cell types, while PC2 captures organ-level differences, indicating that differences between HCs and all other inner ear cell types outweigh differences between organs. However, the separation of all cochlear and utricular cells, even TECs and NSCs, along PC2 suggests that the vestibular and auditory distinction extends beyond the specialized sensory cells. Moreover, the organ-specific separation between HCs and SCs might indicate that despite similar morphological and physiological profiles, auditory and vestibular sensory cells are transcriptionally unique by P1. Alternatively, this separation could be driven by differences in developmental status, with cells from one organ being more mature than the other. However, this seems less likely given that utricular cells are heterochronic in relation to the cochlea, which should result in some utricular cells separating with cochlear cells along PC2 if maturity underlies the majority of the variance.

To begin to identify the specific factors that might drive this distinction, cells were pooled based on organ of origin and compared to determine the unique genes that define vestibular and auditory identity (Fig. 9c). By identifying genes that were expressed in all cells from one organ but no cells from the other, we found 137 genes specifically expressed within the utricle and 53 genes specifically expressed within the cochlea (Fig. 9c; Supplementary Data 2). In addition, 71 genes that failed to reach significance and were detected in all cells from both cochlea and utricle are likely to be shared between the two organs (Fig. 9c; Supplementary Data 2). As a final demonstration of the utility of comparing single cells from different organs, transcriptional profiles between utricular and cochlear HCs were examined to identify factors that were unique to each cell type (Fig. 9d; Supplementary Data 2). Results indicated expression of unique candidates in each set of cells, suggesting factors that could play a role in specification of HC organ types.

## Discussion

The sensory epithelia of the inner ear are complex structures composed of limited numbers of multiple types of highly specialized HCs and SCs. The specification of these individual cell types requires unique transcriptional programs that must be successfully executed both during initial development and in any naturally occurring or induced regenerative process. However, most identified cell-type-specific genes show uniform expression within the most encompassing classes, such as *Gfi1* expression in all HC types. Since previous work has used one or more of these encompassing genes as the basis for isolation and pooling of similar cell types, it has been difficult to identify HC and SC subtypes. Here we have utilized single-cell RNA-Seq technology to generate transcriptional profiles for individual HCs and SCs from both the utricle and cochlea and at different stages of differentiation. The results identify subtypes of both HCs and SCs and provide lists of known and novel genes that are unique or significantly enhanced in each cell population. Subsequent functional assays will be required to determine which of these candidates might act as instructive factors for the specification of unique subtypes.

As a result of a high degree of heterochrony at P1, the analysis of transcriptional profiles for cell types from the utricle revealed cells actively transitioning between fates. The ability to model these transitions along a pseudo-time trajectory allows for the construction of the transcriptional network required to generate unique inner ear cellular phenotypes. Moreover, the observation of differentiating HCs at the edges of the sensory epithelium suggests that the concept of a strict prosensory population within the otocyst that becomes uniquely specified to develop as HCs and SCs needs to be reconsidered, as this hypothesis does not predict the existence of a transitional region where cells may decide between a sensory and non-sensory fate. Whether true NSCs located outside the transitional region are able to give rise to HCs will require further testing through lineage-tracing experiments, but is supported by various genetic manipulations that generate HCs within otherwise non-sensory domains^31^,^54^.

In summary, we have shown that single-cell RNA-Seq is a feasible approach for generating transcriptomic profiles for cells from some of the smallest, most-specialized vertebrate organs. The future application of single-cell profiling to the inner ear or other systems with limited numbers of diverse cell types should make it possible to generate comprehensive transcriptional networks that describe each step in the development of specialized phenotypes. The identification of these networks will be essential for our understanding of development processes and the creation of regenerative therapies.

## Methods

### Animals

CD-1 mice were obtained from Charles River; the Tg(*Lfng-EGFP*)HM340Gsat BAC transgenic mouse line (*Lfng*^*EGFP*^) was generated by the GENSAT project^25^ and was obtained from A. Doetzlhofer; B6;129S6-*Gt(ROSA)26Sor*^*tm9(CAG-tdTomato)Hze*/^ J mice (*R26R*^*CAG-tdTomato*^) were generated by H. Zeng^27^ and were obtained from Jackson Laboratories; and *Gfi1*^*Cre/+*^ mice (*Gfi1*^*Cre*^) were generated and generously provided by L. Gan^26^. These three lines were crossed to generate mixed-background *Lfng*^*EGFP*^; *R26R*^*CAG-tdTomato*^; *Gfi1*^*Cre*^ mice that express GFP in all inner ear sensory patches and tdTomato in HCs^26^,^55^. P1 mice of either sex were used for all experiments. *Lfng*^*EGFP*^; *R26R*^*CAG-tdTomato*^; *Gfi1*^*Cre*^ mice were used for single-cell RNA-Seq experiments and immunolabelling where indicated, both *Lfng*^*EGFP*^ and P1 *Lfng*^*EGFP*^; *R26R*^*CAG-tdTomato*^; *Gfi1*^*Cre*^ mice were used for single-cell qPCR experiments and CD-1 mice were used for immunolabelling. All experiments were conducted in accordance with the NIH animal use protocol, 1262-12. Genotyping primer sequences are provided in Supplementary Table 2.

### Quantification of fluorescent protein expression

High-resolution ( × 40/1.4 numerical aperture objective) confocal images were obtained of fixed utricles and cochleae from *Lfng*^*EGFP*^; *R26R*^*CAG-tdTomato*^; *Gfi1*^*Cre*^ mice on a Zeiss LSM 710. For utricles, the native GFP and tdTomato intensity was measured at the apical surface of every HC and an equal number of SCs (*n*=3 utricles). For cochleae, apical surface intensity measurements were made for native GFP, tdTomato, as well as the fluorophore marking Myo7a immunolabelling in 400 HCs and 25 SCs in regions 20, 50 and 80% from the base of the cochlea (*n*=3 cochleae). The intensity distributions and percentage of fluorescent cells is described in Supplementary Figs 1 and 2.

### Cell isolation and FACS purification of cochlear SCs

For each utricular cell capture, four to five *Lfng*^*EGFP*^; *R26R*^*CAG-tdTomato*^; *Gfi1*^*Cre*^ mice from one to three litters were killed at P1. The vestibular labyrinth was removed from each ear (*n*=8–10 utricles per IFC capture), and utricles were isolated in ice-cold DMEM/F-12 (Life Technologies). The roof and non-sensory epithelium were trimmed away with forceps, and otoconia were removed with a hair. A small strip of transitional epithelium at the border between sensory and non-sensory epithelium was included to be sure that all cells within the sensory epithelium were isolated. The presence of GFP and tdTomato signal was verified with a fluorescence stereomicroscope, and utricles were transferred to a solution of DMEM/F-12 containing 0.2 mg ml^−1^ thermolysin (Sigma) and 10 kunitz ml^−1^ DNase I (Stem Cell Technologies) for 10 min at 37 °C. The organs were then returned to ice-cold DMEM/F-12 where the epithelium was separated from the underlying mesenchyme. The delaminated epithelia were collected in a curette and transferred to 0.5 ml of Accutase (Innovative Cell Technologies) in a 1.5-ml tube on a heat block at 37 °C. After 10 min, the epithelia were gently triturated with a 200-μl pipette, returned to the heat block for 5 min and then gently triturated with a blunted 26-G needle. A total of 0.5 ml of ice-cold DMEM/F-12 was added to the Accutase, and the dissociated cells were pelleted at 300*g* in a swinging bucket centrifuge for 5 min at 4 °C. The supernatant was aspirated until only 15–20 μl remained, and the cells were resuspended and placed on ice until capture. To assess cell viability after the isolation procedure, cells from wild-type mice were isolated, labelled with a LIVE/DEAD assay (Life Technologies, Supplementary Fig. 4a) and cultured successfully for 48 h.

For each cochlear cell capture, a single *Lfng*^*EGFP*^; *R26R*^*CAG-tdTomato*^; *Gfi1*^*Cre*^ mouse was killed at P1 and the inner ears were removed and placed in ice-cold DMEM/F-12. Following removal of the cochlear capsule, tissue was placed in DMEM/F-12 with 0.2 mg ml^−1^ thermolysin and 10 kunitz ml^−1^ DNase I and incubated at 37 °C for 10 min (*n*=1–2 cochleae per IFC capture). Surrounding tissue and underlying mesenchyme was removed from the cochlear epithelium, and then most non-sensory epithelial tissue surrounding the organ of Corti was mechanically removed with forceps and a sapphire knife (WPI). Sensory-enriched epithelia were transferred to a 1.5-ml tube with 0.25% trypsin-EDTA (Life Technologies) and incubated at 37 °C for 15 min, with trituration (50 times with a 200-μl pipette set to 150 μl) at 5-min intervals. Dissociated cells were passed through a 40-μm strainer (BD) before being pelleted at 300*g* and resuspended in 10–15 μl in 10% fetal bovine serum (FBS) (Life Technologies) in DMEM/F12.

Cochleae from *Lfng*^*EGFP*^ mice were used for FACS purification of GFP+ SCs before capture. Briefly, cochleae were dissected and collected in a 1.5-ml tube (*n*=6–8 cochleae per IFC capture). They were then incubated in 0.05% crude trypsin (Worthington) in CMF-PBS (Life Technologies) at 37° for 8 min. Excess trypsin solution was removed and four volumes of 5% FBS in DMEM/F12 was added to stop the digestion. The tissue was then triturated for 2 min and passed through a 40-μm strainer to eliminate residual aggregates. The resulting single-cell suspension was stained with propidium iodide (Life Technologies) to allow for exclusion of dead cells and debris from the samples. Single cells were sorted on a FACSAria flow cytometer (BD Biosciences) with a compensated FITC setting and 488 nm excitation, using a 100-μm nozzle. In neonatal tissue, GFP+ SCs are the brightest population and typically comprised 8–10% of viable cells. A highly stringent gating procedure was applied to collect only the brightest cells. Cells were collected in 20% FBS in DMEM/F12 and stored on ice. After sorting, cells were spun at 200*g* for 10 min and then resuspended in 20% FBS. Schematics of isolation methods are provided in Supplementary Fig. 3.

### Microfluidic capture of single cells and generation of cDNA

Cell capture, lysis, SMARTer-based RT and PCR amplification of cDNA was performed as outlined in the Fluidigm protocol (PN 100–5950 B1) and elsewhere^22^,^23^,^56^. Briefly, after obtaining a single-cell suspension, 10 μl of cells at a final concentration of 2.5 × 10^5^–7 × 10^5^ cells per ml were loaded onto a medium-sized (10–17 μm) IFC. Cell concentration was estimated at a 1:10 dilution using an automated cell counter (Luna). The IFC was placed in the C1 system, where cells were automatically washed and captured. After capture, the chip was removed from the C1, and a 30-μm stack of widefield fluorescence and brightfield images was recorded at each capture site using a × 10/0.4 numerical aperture objective on an inverted Zeiss Axio Observer.Z1 microscope equipped with a motorized stage (see example images in Supplementary Fig. 4d). Little to no crossover of GFP and tdTomato fluorescence was detected on a colocalization plot (Supplementary Fig. 4c). A custom script was written within the Zeiss Zen Blue software to automate this process. Average imaging time for all 96 capture sites was 35 min. A summary of each C1 capture can be found in Supplementary Table 1. After the imaging period, the IFC was returned to the C1 where lysis, RT and PCR were performed automatically within individual reaction chambers for each cell. For RNA-Seq, mixes were prepared from the SMARTer Ultra Low RNA kit (Clontech) according to the volumes indicated in the Fluidigm protocol. For qPCR, mixes were prepared from the Single Cell-to-Ct qRT-PCR kit (Ambion). The thermal cycler within the C1 performs 21 or 18 rounds of PCR amplification to obtain enough material for RNA-Seq or qPCR, respectively. cDNA was manually collected from the output channel of each capture site and stored in a 96-well plate at −20 °C until library preparation. The average time from dissection to cell lysis was ∼3 h for utricle cells, ∼3.5 h for cochlear epithelium cells and ∼5 h for FACS-purified cochlear SCs.

### ERCC RNA standard spike-ins

To test the sensitivity and reproducibility of single-cell RNA-Seq, we added External RNA Controls Consortium (ERCC) RNA spike-in control mix 1 (Ambion) to the cell lysis solution. This mix consists of 92 synthetic polyadenylated messenger RNAs generated from plasmids developed and validated by the ERCC. We tested final spike concentrations of 1:40,000, and 1:20,000 over the course of the 9 C1 captures (Supplementary Table 1). Spikes at a concentration of 1:40,000 were only detectable in one of four captures, and were considered unreliable. Spikes at a concentration of 1:20,000 were detectable in all five captures (see Supplementary Fig. 4e for examples from each capture type). At this concentration, the measured level of spikes was consistent in four of the five captures; however, the spikes from one capture appeared to show degradation, which was likely due to spikes being diluted too early before capture.

### Preparation of pooled cell populations for RNA-Seq

While single cells were capturing on the C1 system, the leftover single-cell suspension was washed twice with Fluidigm Cell Wash Buffer, diluted to a final concentration of 1–2 × 10^5^ cells per ml and then stored on ice. Immediately after lysis was initiated on the IFC, 1 μl of this suspension (that is, 100–200 cells) was taken for preparing a pooled cell tube control with the same lysis, RT and PCR mixes and thermal cycler conditions that were used for single cells (see Supplementary Fig. 4f for isolations in which tube controls were collected). We also obtained a larger pooled population of FACS-purified cochlear SCs (∼2,000 cells) on a separate day. These cells were lysed and RNA was extracted using the Quick-RNA MicroPrep Kit (Zymo Research). Library construction was performed identically as described for single cells below, with the exception that only four barcoded tube control samples were pooled together for sequencing on one lane.

### RNA-Seq library construction

Single cells were selected from the z-stack images of each capture site using stringent guidelines, eliminating any sites with suspected multi-cell or empty captures (Supplementary Fig. 4d). Images for each single cell used in this study are available upon request. Capture sites were excluded if there was any doubt about a single-cell capture. The threshold for positive detection of GFP and tdTomato fluorescence was set just above background to include cells with low levels of fluorescence that may have recently acquired *Lfng* or *Gfi1* promoter activity. Using a low threshold was consistent with the observation that fluorescence intensity was log-normally distributed within the cells (Supplementary Figs 1 and 2). For comparison purposes, some multi-cell and empty capture sites were selected for library preparation and sequencing. These were not included in our analyses of single cells presented here.

The concentration of cDNA obtained from each capture site was measured in duplicate using a PicoGreen assay (Life Technologies) and a Beckman Coulter DTX 880 fluorescence plate reader. cDNA was diluted to a final concentration of 0.1–0.3 ng μl^−1^, then tagmented and tagged with adapter sequences using the Nextera XT DNA Sample Preparation Kit (Illumina), as described in the Fluidigm protocol. The adapter sequences were then used as primer recognition sites for a limited-cycle PCR reaction (12 cycles) in which sequencing primer and unique barcode sequences were added using the Nextera XT DNA Sample Preparation Index Kit (Illumina). Finally, barcoded libraries from 48 cells were pooled and cleaned using AmPure XP beads (Agencourt). Single-cell libraries originating from multiple C1 captures were pooled together to avoid compounding any potential C1 capture bias with sequencing lane bias (Supplementary Table 1).

### Sequencing of libraries and estimation of gene expression

Each collection of 48 pooled single-cell libraries was sequenced on a single-flow cell lane of an Illumina HiSeq 1000 to an average depth of 3.4 M reads using 90 × 90 paired-end reads. The four 100–200-cell tube control libraries from each organ were pooled and sequenced on separate lanes to an average depth of 65 M reads. Reads were de-multiplexed and then aligned to a Bowtie index based on the NCBI-annotated mouse transcriptome (extracted from the 26,583 genes in GRCm38 genome with the corresponding GTF) using Bowtie 2v2.2.3. The sequences and identifiers for enhanced GFP (EGFP) and tdTomato were appended to the genome FASTA and the GTF before creating the index used for alignment. For each cell (library), relative transcript abundances were estimated from the aligned reads using RSEM v1.2.19 (default parameters)^57^,^58^. RSEM estimates transcript abundance in units of transcript per million (TPM). The abundances reported here are at the gene level, which RSEM calculates by summing the estimated transcript abundances for each gene. Since abundance estimates are relative, the 92 ERCC RNA standards were aligned and quantified in a separate Bowtie/RSEM run to avoid suppressing the levels of endogenous transcripts with the exogenous spike-ins (Bowtie index created by appending the sequences for EGFP, tdTomato and ERCC's to GRCm38). Alignment and abundance estimation were carried out on the NIH/Helix Biowulf cluster.

SMARTer chemistry has previously been shown to yield moderate levels of intronic mapping and 3′-bias^29^,^59^,^60^. To examine these metrics, reads were aligned to GRCm38 using HISAT (0.1.5)^61^, and mapping rates and transcript coverage were calculated with RNA-SeQC^62^ and RSeQC^63^, respectively. The percentage of reads mapping to transcriptome (for Bowtie/RSEM), exons, and introns and 3′-bias were all consistent with the referenced publications (Supplementary Fig. 8a–c).

### Cross-sample normalization

Since comparisons of relative abundances can be misleading^64^, we normalized the relative transcript abundance estimates across cells. For this, we employed a commonly used approach available within the DESeq analysis package^65^, which scales each sample by the median of the geometric means across all samples. First, the TPM values for each gene are divided by their geometric mean across the samples. Then the within-sample median of these ratios is used as the normalization factor for each sample (genes with a geometric mean of zero are not included in the median calculation). Cross-sample normalization was performed separately for each analysis shown so that only the cells being compared were normalized with respect to each other. Since the cross-sample-normalized TPMs are not true TPM units, we refer to them as nTPMs to designate normalized relative abundance.

### Limit of detection and data transformation

Analysis of ERCC spike-ins showed that some of the low-concentration transcripts were not detectable in any of the capture sites (Supplementary Fig. 4e, ERCC molecules with 0 mean nTPM). For each isolation with detectable levels of ERCC transcripts, all transcripts became detectable (that is, mean nTPM≠0) at a mean nTPM of ∼1. Therefore, we set 1 nTPM as our limit of detection (LOD), which is comparable to the LOD used in other published reports^22^,^23^,^56^. For all analyses, nTPMs <1 were set to zero, and TPMs >1 were transformed to log_2_(nTPM) space. For the comparisons of average single-cell gene expression to 100–200 cell pooled populations (Fig. 1i; Supplementary Fig. 4f), single-cell expression values were converted to log_2_(nTPM) after averaging.

### Outlier identification

Any cells that appeared unhealthy in the recorded capture site images were excluded from library preparation. Saturation of unique genes detected was used to exclude cells that did not have sufficient sequencing depth. Briefly, the number of unique genes expressed per cell versus aligned reads contributing to counts was determined by down-sampling paired-end reads from representative cells using seqtk as previously described (Supplementary Fig. 8d)^23^. The depth on the saturation curve where 90% of unique genes were detected was used as the cutoff (∼2.5 × 10^5^ total aligned reads per cell). To further identify potentially unhealthy cells with abnormally low expression levels, we passed the cells through the outlier identification function provided in SINGuLAR Analysis Toolset 3.0, Fluidigm's R package for single-cell expression analysis. Outlier identification in SINGuLAR proceeds by trimming low-expressing genes until 95% of the genes that remain are above 1 nTPM in half of the cells. A distribution of combined gene expression is created from these cells, and outliers are considered as cells whose median expression across the identified gene list is below the 15th percentile of the distribution. Using these routines, two utricle cells, one cochlear epithelium cell and nine FACS-purified cochlear SCs were excluded from the analysis (Supplementary Table 1; Supplementary Fig. 8e). Outliers were removed before all analyses described below.

### Correction for batch effects within cochlear cells

PCA analysis showed that utricular cells and FACS-purified cochlear SCs segregated along the first 2–4 PCs almost exclusively by identifiable cell types. However, cochlear cells that were mechanically purified showed separation along PC1 that was heavily influenced by sex-linked genes. Since we only used one pup for each of the two mechanical isolations of cochlear cells (Supplementary Table 1), it appears likely that one pup was male and the other female (sex could not be determined at this age), and these differences were large enough to influence cell-to-cell variation. Since we did not see this effect within the utricular- and cochlear-FACS cells that originated from 4–6 mice per isolation, we suspect that mouse-to-mouse variation suppresses sex differences that would otherwise be seen if only one animal were used per isolation. To correct for these differences within the cochlear cells that were mechanically isolated, we removed isolation batch effects using ComBat within the sva package in R before selecting genes as outlined below^66^.

### Selection of genes for clustering analysis

Custom R scripts were written for clustering analysis of single-cell data. Identification of the top genes with PCA was performed using described methods^22^. Briefly, genes with nTPM>1 in only two or fewer cells or with a cell-to-cell coefficient of variation <0.5 were removed, then PCA was performed on the remaining gene list using the FactoMineR package in R. The number of expressed genes detected for the clustering analyses described throughout the text ranged from 10,268 to 14,295. See Supplementary Data 2 for the number of genes detected for each particular analysis. Genes that were most highly correlated (positively and negatively) with the first 2–4 PCs were then selected for subsequent clustering analyses. The number of genes selected in this manner was determined empirically by performing PCA and clustering with increasing numbers of selected genes and identifying the minimum number of genes that best captured the heterogeneity among the cells (number of genes chosen for each analysis indicated in the figure legends). The correlations between selected genes and PCs were all deemed significant with a one-way analysis of variance (*P*<0.05) performed with the dimdesc function in FactoMineR.

### Clustering of single cells

*K*-means clustering has been shown to be an effective approach for clustering cells and genes for single-cell RNA-Seq data^67^. Cells and genes were clustered with *k*-means clustering using the cluster package in R. To determine the optimal number of clusters, we calculated gap statistics^68^ for 1–15 clusters and selected the minimum cluster number at which the ‘gap' was no more than one s.e. away from the first local maximum. Hundred Monte Carlo samples were drawn from the reference distribution for calculating the gap statistic. *K*-means clustering was then performed with the identified cluster number. Clustered cells and genes were functionally ordered and plotted on heatmaps using the NMF package in R.

### Differential expression analysis and ordering of cells

Differential expression testing between selected sample groups was performed within Monocle^32^, which fits a generalized additive model (GAM) to the genes using gene expression as the response variable and the designated sample group as the predictor. Monocle then tests for differential expression between the fit and a reduced model (no sample groups) using an approximate likelihood ratio test. A uninormal GAM family was used for the log-transformed nTPM values. Since some genes showed zero expression within a group, a small amount of random noise was added to the data set to prevent a s.d. of zero in the GAM estimation procedure. Genes with nTPM>1 in only two or fewer cells within each group were removed before running differential expression analysis. The number of expressed genes detected for the differential expression tests described throughout the text ranged from 7,058 to 15,102. See Supplementary Data 2 for the number of genes detected for each particular analysis. Genes with an FDR<0.05 after the Benjamini–Hochberg correction of the likelihood ratio test *P* values were considered significant. Sample groups were selected based on clustering results as indicated in the text. For tests of differential expression between *Tectb*+ and *Tectb*− cells, we compared cells with log_2_(nTPM)>10 with those with log_2_(nTPM)=0 (*n*=14 cells).

Due to high levels of technical noise in single-cell RNA-Seq data, only a portion of differentially expressed genes can be identified as biologically variable^28^. Biological variability can be distinguished from technical noise using ERCC spike-ins; however, we did not have uniform or detectable levels of spike-ins in all of our cells. Therefore, we report only a refined list of differentially expressed genes in the Results, which is limited to those genes that showed the highest enrichment within each group using two criteria. First, we eliminated genes that were not detected (above LOD) in ≥50% of cells in the relevant group. Second, we determined the degree to which a gene was exclusively expressed within a particular group of cells by calculating group specificity scores^69^ with a modification of the cummeRbund package available from Bioconductor. For this, we calculated the geometric means of the genes within each group, excluding cells with nTPM=0 from the calculation, and the resultant geometric means were converted to log_2_(nTPM). CummeRbund uses these values to create a probability vector of each gene's expression across the sample groups, and then calculates specificity as the Jensen–Shannon distance between the calculated probability vector and the ideal probability vector in which a gene is only expressed in one group. Specificity scores range from 0 to 1, with 0 being least specific and 1 being perfectly specific. Within the list of genes that show significant differential expression (FDR<0.05, Supplementary Data 2), gene specificity scores that are greater in one group compared with the other indicate that a gene is expressed at higher levels in the group with the higher score. We used arbitrary specificity score thresholds of 0.5 and 0.6 (for comparisons between two groups and three groups, respectively) to filter the lists of differentially expressed genes. Violin plots showing the distribution of gene expression within each group were generated with the ggplot2 package in R.

Ordering of utricular cells along a differentiation trajectory (pseudo-time) was performed in Monocle. A natural spline with three effective degrees of freedom was used to model gene expression as a smooth, nonlinear function over pseudo-time (blue curve fits in Fig. 4), and genes whose expression showed significant variation over pseudo-time were identified with an approximate likelihood ratio test comparing this model against the reduced model of no pseudo-time dependence. Genes were selected at an FDR <0.05 and clustered by similarities in kinetic trends using Monocle. The number of clusters (*k*=4, Fig. 5c) was selected empirically based on the maximum cluster number that produced average trends that appeared dissimilar.

### Inter-organ comparisons

For the inter-organ comparisons described in Fig. 9, we used the following cell clusters: for utricle, TEC, SC.ii and HC.iii–iv; for cochlea NSC.i–ii, SC and HC. We excluded the transitional utricle cell clusters (SC.i and HC.i–ii) since they represented combinations of cell types that were already present in the analysis. We excluded FACS-purified SCs, so that we only compared cells that were isolated using similar methods. Thus, a total of 202 utricular and cochlear cells were used for the analyses. Cells were cross-sample normalized, and expressed genes were identified as described above. Three-dimensional PCA plots were generated with the rgl package in R. Differential expression analysis and subsequent calculation of specificity scores were performed as described above.

### Validation of qPCR primer set assays

A total of 96 DELTAgene qPCR gene expression assays, consisting of forward and reverse qPCR primers, were purchased from Fluidigm. Thirty of the 96 assays were used for single-cell qPCR analysis in this study. qPCR primer sequences are provided in Supplementary Table 3. The remainder either did not pass our validation criteria or were used for other experiments. DELTAgene assays were validated against mouse universal cDNA. Briefly, 20 ng of universal cDNA was subjected to 18 cycles of preamplification (identical to the C1 workflow) using a pooled mix of all 96 DELTAgene assays and TaqMan PreAmp Master Mix (Cells-to-C_T_ kit, Life Technologies) as outlined in the Fluidigm protocol (PN 68000088 Rev G1). Residual primers were removed from the reaction product with 40 units of Exonuclease I (New England BioLabs Inc.). To determine primer efficiencies for all 96 primer sets, we made 8 threefold dilutions of the preamplification product and tested them in triplicate on a 96.96 Dynamic Array IFC using a Fluidigm BioMark HD microfluidics-based qPCR system. Primer efficiency was calculated with the formula: 
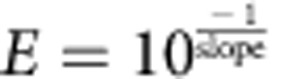
, where slope represents the slope of a linear regression fit to the average standard curve^70^. Primer sets with efficiencies ±0.2 from the ideal efficiency of 2 or for which we detected multiple peaks on a melt curve were excluded from further analysis (Fig. 3). Sixteen of the 96 assays failed to pass these criteria.

### Single-cell qPCR

Gene expression levels for the 30 selected DELTAgene assays within single-cell cDNA was measured with qPCR on a 96.96 Dynamic Array IFC using the Fluidigm BioMark HD system. cDNA from single cells was selected for qPCR in the same way as it was selected for RNA-Seq. A total of 118 single cells from three C1 captures were profiled using three Dynamic Array IFCs. Gene expression between different C1 captures or qPCR BioMark runs was well correlated, indicating that there was little technical variation between runs (Fig. 3). We used a conservative Ct of 24 as LOD based on published guidelines^70^. Gene expression was defined on a log_2_ scale as: log_2_ expression=LOD*−*Ct, as described in the Fluidigm manual (PN 100–5066 E3). All analyses of single-cell qPCR data, including PCA and hierarchical clustering, were performed with default parameters in SINGuLAR.

### Immunohistochemistry and *in situ* hybridization

Utricles and cochleae were fixed in fresh 4% paraformaldehyde in PBS for 30 min at room temperature or Shandon Glyo-Fixx (ThermoFisher Scientific) overnight at 4 °C. After fixation, specimens were washed in PBS then permeabilized and blocked for 1 h at room temperature in PBS with 0.2% Triton X-100 (PBS-T) and 10% normal goat serum (Vector Labs) or 10% normal horse serum (Vector Labs). Samples were then incubated in the appropriate primary antibodies in PBS-T with 2% normal goat serum or normal horse serum overnight, followed by three rinses in PBS-T and labelling with AlexaFluor-conjugated secondary antibodies (1:1,000, Life Technologies) in PBS-T for 3 h at room temperature. Where indicated, AlexaFluor-conjugated phalloidin (5 U ml^−1^, Life Technologies) and/or 4,6-diamidino-2-phenylindole (1:2,000, Life Technologies) were included with the secondary antibodies to detect F-actin and nuclei. Organs were rinsed in PBS three times and mounted in SlowFade (Invitrogen) or Fluoromount-G (Southern Biotech). Specimens were imaged using a Zeiss LSM 710 confocal microscope. For immunohistochemistry and *in situ* hybridization of cochlear sections, fixed inner ears were embedded in Optimal Cutting Temperature compound (OCT) and sectioned in a cryostat set to 10–12 μm.

*In situ* hybridization was performed using previously published methods^71^. Briefly, a Mia1 cDNA fragment was subcloned from Origene MC200907 into pBluescript by KpnI/NotI digestion. RNA antisense probes were transcribed from the linearized vector (KpnI or NotI, for antisense and sense control). For immunostaining following *in situ* hybridization, slides were washed 3 × 10 min in TBS-T before the standard immunofluorescence procedure detailed above.

The following antibodies were used: rabbit anti-Gata2 (1:200; ThermoFisher Scientific, PA1–100), rabbit anti-Sall2 (1:200; Sigma, HPA004162), rat anti-R-cadherin (1:50; Developmental Studies Hybridoma Bank, MRCD5), mouse anti-N-cadherin (1:200; BD Biosciences, 610920), rabbit anti-Myosin VIIA (1:1,000; Proteus BioSciences, 25–6791), goat anti-Contactin-1 (1:200; Neuromics, GT15055), mouse anti-Pou4f3 (1:500; Santa Cruz Biotech, sc-81980), goat anti-Sparcl1 (1:200; R&D Systems, AF2836), rabbit anti-Rasd2 (1:200; ThermoFisher Scientific, PA5–20439), rabbit anti-Pcp4 (1:100; Santa Cruz Biotech, sc-74816), goat anti-Annexin A4 (1:200; R&D Systems, AF4146), goat anti-Sox2 (1:200; Santa Cruz Biotech, sc-17320), goat anti-Pvalb (1:200; Swant, PVG-214), goat anti-Xirp2 (1:200; Santa Cruz Biotech, sc-83128), goat anti-Fscn2 (1:200; Everest Biotech EB08002), goat anti-Ocm (1:200; Santa Cruz Biotech, sc-7466), goat anti-Clusterin (1:100; R&D Systems, AF2747), rabbit anti-Tectb (1:1,000; generous gift from G. Richardson), mouse anti-Pou3f3 (1:100; Santa Cruz Biotech, sc-6028-R), mouse anti-E-cadherin (1:200; BD Biosciences, 610181), mouse anti-Spectrin (1:50; Millipore, MAB1622), rabbit anti-Pvalb3 (now Ocm; 1:200; generous gift from S. Heller) and mouse anti-Mia1 (1:100, R&D Systems, MAB20501).

## Additional information

**Accession codes:** RNA-Seq data have been deposited in the NCBI GEO database under accession code GSE71982.

**How to cite this article:** Burns, J. C. *et al.* Single-cell RNA-Seq resolves cellular complexity in sensory organs from the neonatal inner ear. *Nat. Commun.* 6:8557 doi: 10.1038/ncomms9557 (2015).

## Supplementary Material

Supplementary Information Supplementary figures 1-8 and Supplementary Tables 1-3.

Supplementary Movie 1 3-D PCA plot of utricular and cochlear cells. Movie shows rotation of 3-D PCA plot in Fig. 9a.

Supplementary Data 1 Top genes identified by PCA analysis.

Supplementary Data 2 Results of all trajectory and differential expression analyses performed in Monocle.
