# Supplementary material for: Single-cell RNA-Seq resolves cellular complexity in sensory organs from the neonatal inner ear
Source: Nat Commun. 2015 Oct 15;6:8557. doi: 10.1038/ncomms9557 (PMC4634134; doi:10.1038/ncomms9557)
Supplement: Supplementary Information — Supplementary figures 1-8 and Supplementary Tables 1-3. [file ncomms9557-s1.pdf]

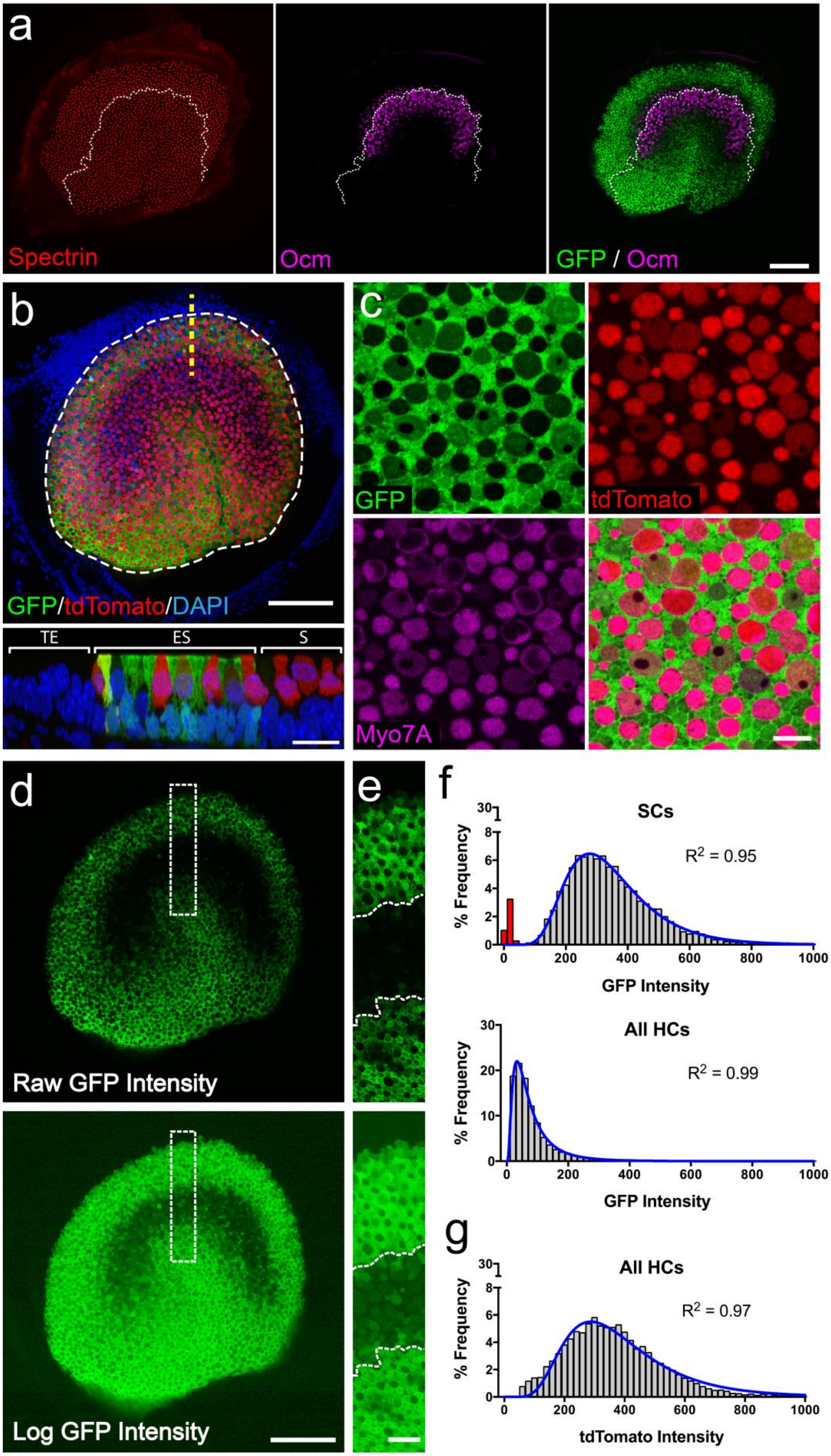
a
Spectrin
Ocm
GFP / Ocm
b
GFP/tdTomato/DAPI
TE
ES
S
c
GFP
tdTomato
Myo7A
d
Raw GFP Intensity
Log GFP Intensity
e
f
SCs
% Frequency
GFP Intensity
R2 = 0.95
All HCs
R2 = 0.99
g
All HCs
tdTomato Intensity
R2 = 0.97

Supplementary Figure 1. Analysis of GFP and tdTomato expression in the utricle of P1 *Lfng^{EGFP}*; *R26R$^{CAG\text{-}tdTomato}$*; *Gfi1^{Cre}* mice. a, Confocal images of a utricle from a P1 *Lfng^{EGFP}* mouse. The striola is a crescent-shaped region located largely on the medial side of the line of HC polarity reversal (white dashed line). To identify the line of polarity reversal, HC cuticular plates were labeled with anti-spectrin (red). Comparison with GFP expression (green) illustrates the position of the reversal line on the lateral side of the region of low GFP expression. To confirm that low GFP expression corresponds with the striola, the calcium binding protein oncomodulin, which is expressed specifically in HCs within the putative striola, was visualized with an antibody against oncomodulin (Ocm)[73]. The merged GFP/Ocm image demonstrates the precise location of Ocm+ HCs within the region of low GFP expression. **b,** Top: confocal image of a whole mount utricle from a P1 *Lfng^{EGFP}*; *R26R$^{CAG\text{-}tdTomato}$*; *Gfi1^{Cre}* mouse. Nuclei have been counterstained with DAPI. Bottom: orthogonal section through the confocal stack (yellow line on whole mount image indicates section location). GFP is specifically expressed within the extrastriolar sensory epithelium (ES) but is not evident in the striolar sensory epithelium (S) or in the non-sensory transitional epithelium (TE). GFP and tdTomato fluorescence is from fixed fluorescent protein and has not been amplified with antibody labeling. **c,** High resolution confocal images of an extrastriolar region from the utricle in **(b)**. All SCs are GFP+ while some HCs also express GFP at low levels. HCs are additionally labeled with antibodies to myosin VIIA (Myo7a, purple). All Myo7a+ HCs express tdTomato at a level above background. Less than 1% of Myo7a+ cells express tdTomato at low levels (less than 4-times above background). tdTomato fluorescence intensity in all Myo7a+ HCs (6661 HCs in total) from three P1 *Lfng^{EGFP}*; *R26R$^{CAG\text{-}tdTomato}$*; *Gfi1^{Cre}* mouse utricles was measured to determine the percentage of tdTomato+ HCs. **d,** Confocal images of raw and log-scale GFP intensity from the utricle in **(b)**. Many of the striolar cells that appear to be GFP– on a linear scale express GFP at a level above background (area surrounding the sensory epithelium) when displayed on a log-scale. **e,** Zoomed regions from the boxes in **(d)** show that striolar HCs express GFP at very low levels that are detectable above background on a log-scale. However, GFP in some striolar SCs is still indistinguishable from background, even on a log scale. **f,** Histograms of measured GFP intensity within SCs and HCs. GFP intensity was measured in all Myo7a+ HCs (i.e. striolar and extrastriolar) and in a comparable number of SCs with detectable levels of GFP in the extrastriola (n=3 utricles). GFP intensity was also sampled from a subset of striolar SCs (regions selected based on absence of Myo7a and tdTomato intensity). Measurements from striolar SCs are shown in red bars on the histogram. The distribution of GFP in HCs and extrastriolar SCs is log-normally distributed (blue lines show fits of data to a log-normal equation along with corresponding R^2 values). Unlike in HCs, GFP intensity in striolar SCs comprises a discontinuous peak centered near background on the plot. **g,** Histogram of tdTomato intensity in HCs. tdTomato also appears to be best fit by a log-normal equation. Scale bars: **a**, **b (top)**, **d,** 100 μm; **c**, 10 μm; **b (bottom)**, **e**, 20 μm.

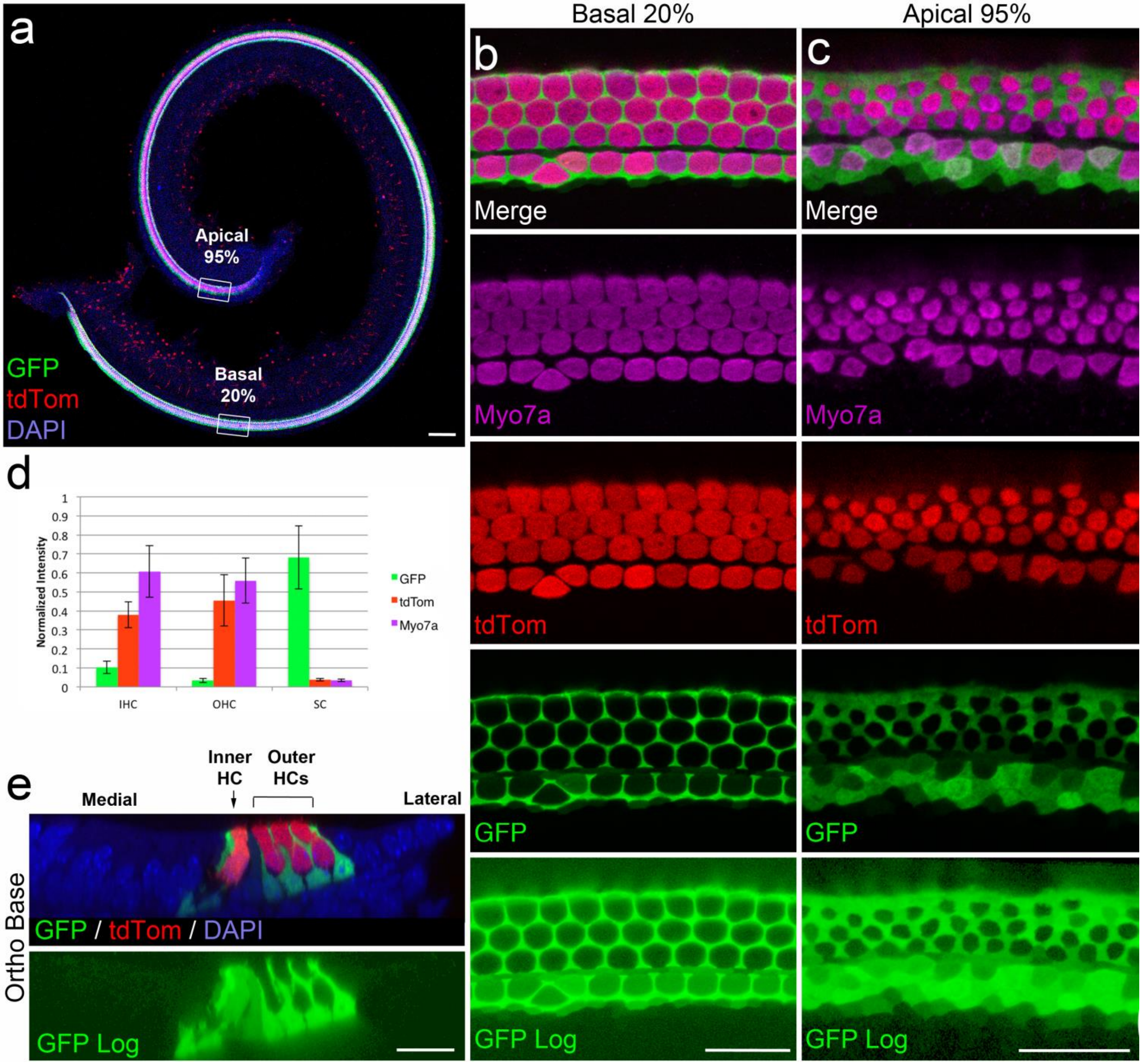

Supplementary Figure 2. Analysis of GFP and tdTomato expression in the cochlea of P1 *Lfng^{EGFP}*; *R26R$^{CAG\text{-}tdTomato}$*; *Gfi1^{Cre}* mice. **a,** Low-magnification confocal image of the cochlea from a P1 *Lfng^{EGFP}*; *R26R$^{CAG\text{-}tdTomato}$*; *Gfi1^{Cre}* mouse. The organ of Corti is visible as a band of tdTomato-expressing HCs and GFP-expressing SCs amongst all other cochlea cells (counterstained with DAPI). tdTomato+ cells visible outside of the sensory region are located in the underlying mesenchymal layer and are removed prior to dissociation and capture (see Supplementary Fig. 3). **b**, High magnification view of the organ of Corti from a region located at 20% from the base of the cochlea (boxed in a). Fewer than 1% of myosin VIIA+ (Myo7a+) HCs were tdTomato–, and fewer than 1% of SCs were tdTomato+ (Myo7a– cells, n = 3 cochleae). High GFP expression is visible within all SCs, except the inner pillar cells, which delineate the border between the inner and outer HC domains. Weaker GFP expression can be observed within HCs (particularly a subset of inner HCs), inner pillar cells, and non-sensory cells along the border of the organ of Corti when intensity values are log-transformed. **c,** The specificity of GFP is somewhat less specific in the very apical

95% of the organ of Corti, with some HCs and surrounding non-sensory cells having GFP expression at or above that of nearby SCs. **d,** Summary of normalized intensity measurements taken from HCs within a 500 µm span at regions located 20, 50, and 80% from the cochlear base for GFP, tdTomato, and the AlexaFluor-647-conjugated secondary antibody used for detecting Myo7a immunoreactivity. The mean ± s.d. of all the regions combined is shown. Inner HCs had a slight elevation in GFP intensity over background. By comparison, sampled SCs from the same regions had high GFP intensity, but lacked tdTomato or Myo7a intensity above background. **e**, Relative expression of GFP within the inner SCs and inner pillar cells, even at the basal turn, can be observed in the axial orthogonal view when intensity values are log-transformed. Scale bars: **a**, 100 μm; **b**, **c**, **e**, 20 μm.

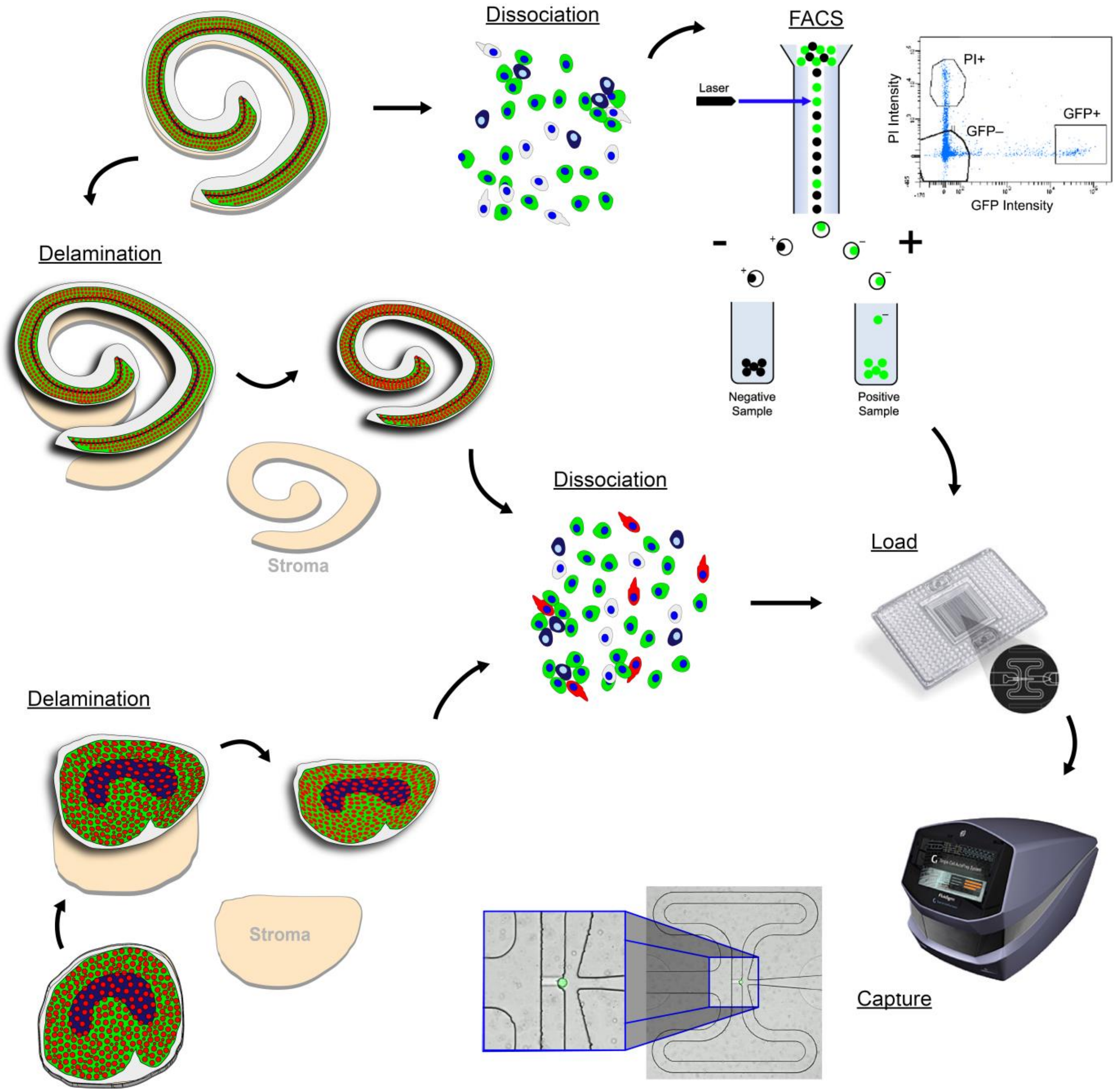

Supplementary Figure 3. Protocol for isolation of single cells from the mouse inner ear. Diagrams of the methods used for isolation of utricular and cochlear sensory epithelial cells from the mouse inner ear. For mechanical purification, utricular and cochlear sensory epithelia were separated from underlying stromal (mesenchymal) cells using an enzymatic treatment. Surrounding non-sensory epithelium was then dissected away using a stereomicroscope equipped with fluorescence to visualize expression of GFP. Some transitional cells at the edge of the border between the sensory and non-sensory epithelium (Greater and Lesser Epithelial Ridges in the cochlea, TEC cells in the utricle) were included in the isolation. Following dissection, epithelial sheets were treated with Accutase or Trypsin followed by mechanical dissociation to yield a single cell suspension containing GFP+, tdTomato+ and non-fluorescent cells. Approximately 2000-4000 cells from each isolation were loaded onto an IFC chip and processed for single cell capture. Individually captured cells were imaged with epifluorescence microscopy prior to lysis and mRNA extraction. To further enrich for cochlear SCs, FACS was used to sort GFP+ cells from *Lfng*^{EGFP} mice for three of the C1 captures in this study. See Methods for further details on the isolation procedures.

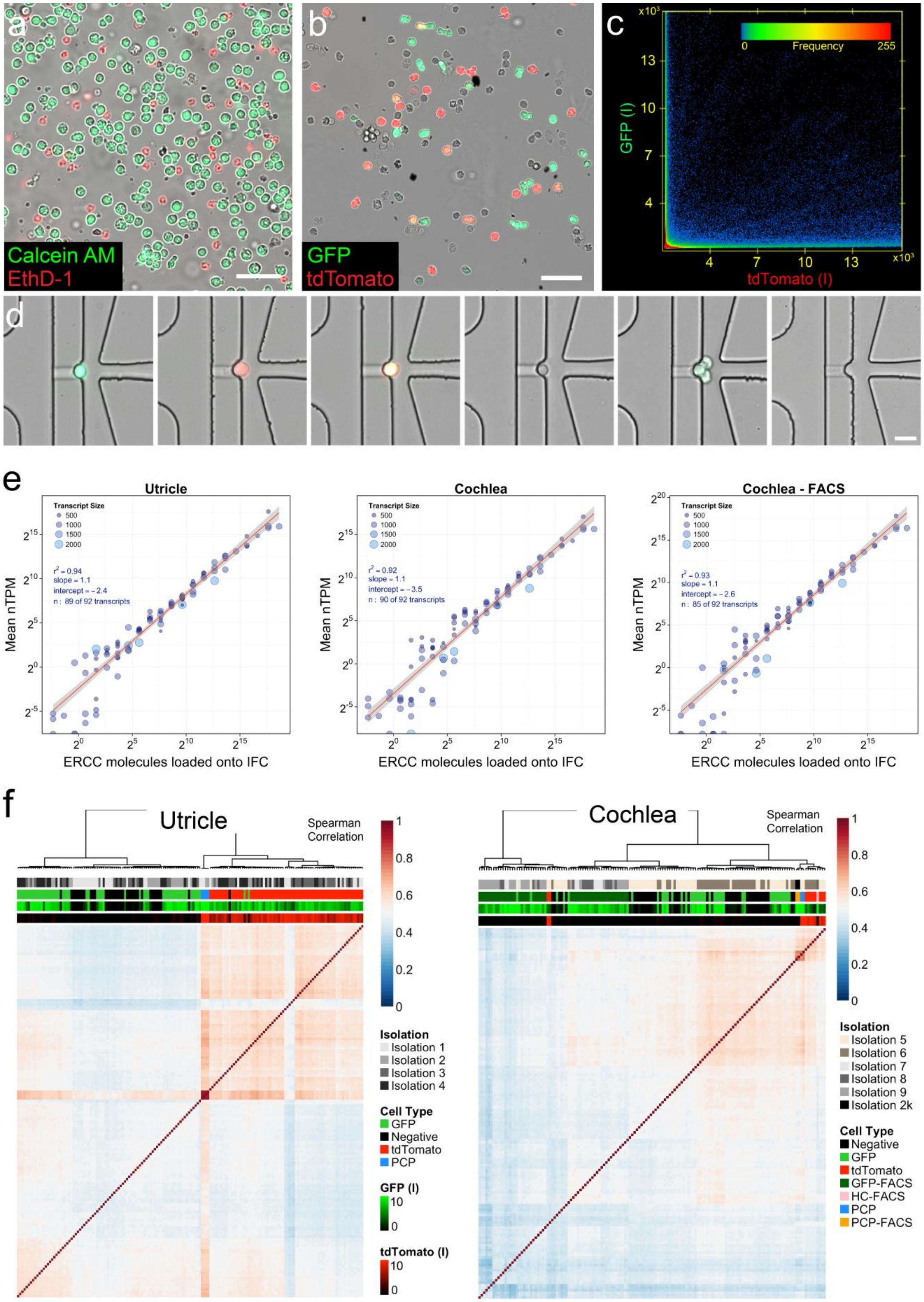

Supplementary Figure 4. Single-cell capture, sensitivity of transcript detection, and correlations in gene expression. a, Widefield image of utricular epithelial cells on a glass coverslip. Cells were isolated from wild-type mouse utricles, dissociated, and labeled with a LIVE/DEAD viability

kit. Calcein AM (green) labels live cells while ethidium homodimer-1 (EthD-1, red) labels nuclei of unhealthy or dead cells with disrupted membranes. The majority of cells are viable after dissociation (see Methods for protocol). **b,** Sensory epithelial cells isolated from *Lfng*^{EGFP}; *R26R*$^{CAG\text{-}tdTomato}$; *Gfi1*^{Cre} mice. SCs are GFP+/tdTomato– (green), HCs are either GFP+/tdTomato+ (yellow) or GFP–/tdTomato+ (red) and TECs are GFP–/tdTomato–. Note that striolar SCs and HCs express GFP at or just above the level of background (see Supplementary Fig. 1). **c,** Co-localization plot for GFP and tdTomato fluorescence intensity in images of cells from *Lfng*^{EGFP}; *R26R*$^{CAG\text{-}tdTomato}$; *Gfi1*^{Cre} mice. GFP and tdTomato show little to no bleed-through with the fluorescence filters used for capturing images. The same imaging parameters used for collecting the data in **(c)** were used for imaging of the capture sites on the C1 IFC's **(d)**. **d,** Widefield images of different types of captures on a C1 IFC, including a single GFP+ cell, a single tdTomato+ cell, a single GFP+/tdTomato+ cell which appears yellow, a single GFP–/tdTomato– cell, a capture site containing three cells, and an empty capture site. **e,** Bubble plots of cross-sample-normalized transcript abundance (nTPMs) versus number of ERCC transcripts in the lysis mix (1:20,000 dilution) for representative captures of cells from utricular (50 cells), cochlear (64 cells), or FACS-purified cochlear (52 cells) isolations. The size of the circles indicates the transcript lengths for the ERCC spike-ins. For each type of isolation, the spike-ins show a linear response ($r^2 \geq 0.92$) over 20, two-fold increases in transcript concentration. **f,** Matrices of the linear correlation (Spearman's *r*) in gene abundance between all samples derived from utricle or cochlea. TPMs were cross-sample normalized and $\log_2$-transformed (LOD=1) as described in the Methods prior to computing the correlation coefficient. Samples have been clustered with unsupervised hierarchical clustering. In general, cells of the same type cluster together, and the isolation from which the cells were obtained appears to have little affect on the correlation trends. Pooled cell populations (PCPs) show the strongest between-sample correlations. In contrast, the correlation coefficients between single cells are lower. Amongst single cells, HCs are most correlated, indicating that more differentiated cell types are less variable than undifferentiated cells that have not committed to a particular fate. In addition, cochlear SCs purified with FACS are poorly correlated with mechanically purified SCs. Thus, the isolation protocol may alter global gene expression patterns and should be taken into account when comparing gene expression across samples obtained using different methods. Scale bars: **a**, **b**, 50 μm; **d**, 20 μm.

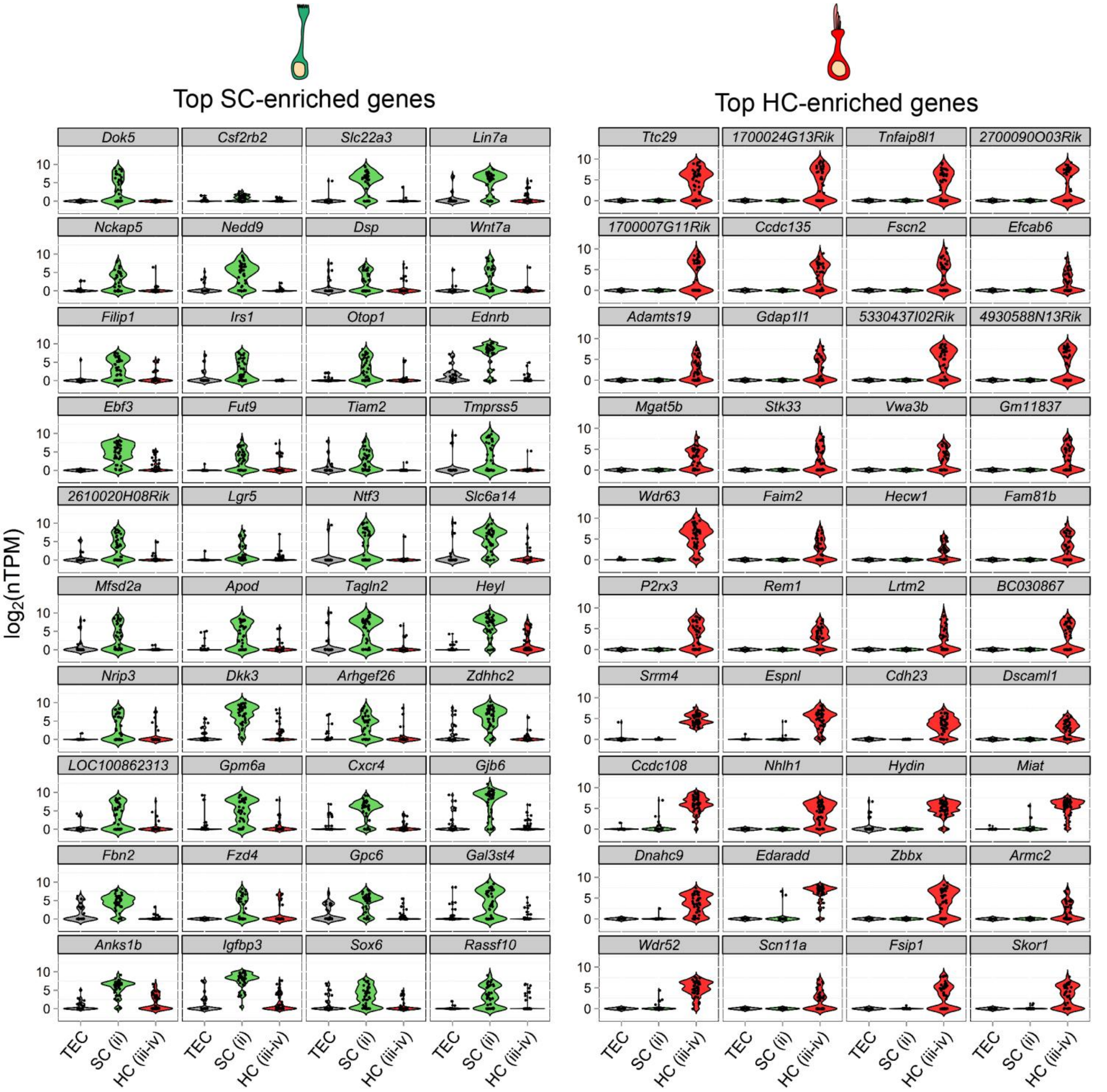

Supplementary Figure 5. Identification of SC- and HC-specific genes in P1 utricle. Violin plots of the top 48 genes (ranked by cell specificity score) expressed specifically or predominantly in SC.ii as compared to TECs and HC.iii-iv (left), and in HC.iii-iv as compared to TECs and SC.ii. Significant differences (FDR<0.05) in gene expression between groups were identified with Monocle prior to calculating specificity scores. Cell groups that were transitioning between states (SC.i, HC.i and HC.ii) were excluded from this analysis to avoid contamination from transitional genes. See Fig. 2 for cell group designations.

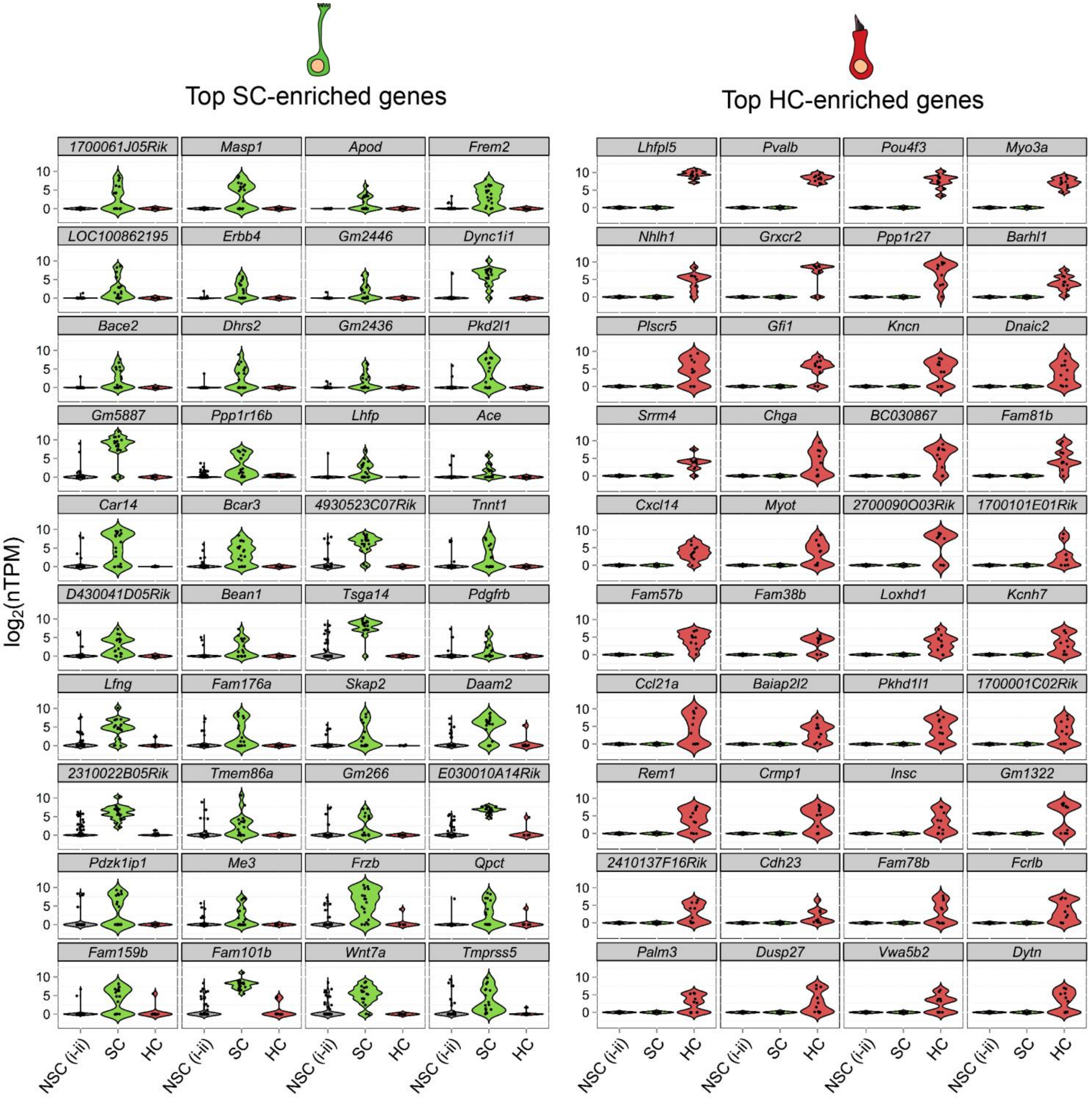

Supplementary Figure 6. Identification of SC- and HC-specific genes in the P1 cochlea. Violin plots for the top 48 genes (ranked by cell specificity score) expressed specifically or predominantly in cochlear SCs as compared with the NSC.i-ii or HCs (left), and in HCs as compared with NSC.i-ii or SCs (right). Significant differences (FDR<0.05) in gene expression between groups were identified with Monocle prior to calculating specificity scores. See Fig. 7 for cell group designations.

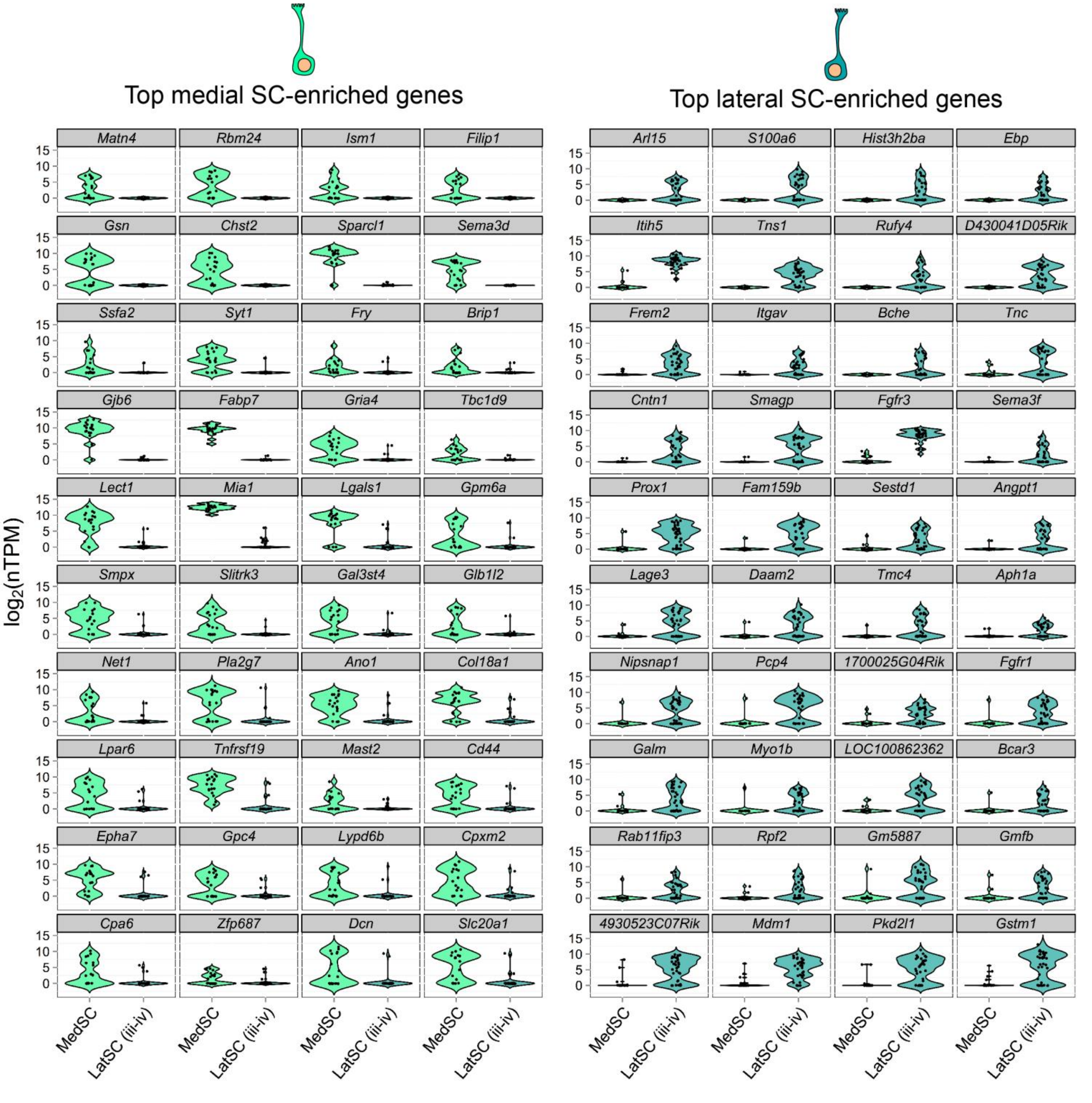

Supplementary Figure 7. Identification of medial-SC-specific genes in the P1 cochlea. Violin plots for the top 48 genes (ranked by cell specificity score) expressed specifically or predominantly in cochlear MedSC (left) and LatSC.i-ii (right). Significant differences (FDR<0.05) in gene expression between groups were identified with Monocle prior to calculating specificity scores. See Fig. 8 for cell group designations.

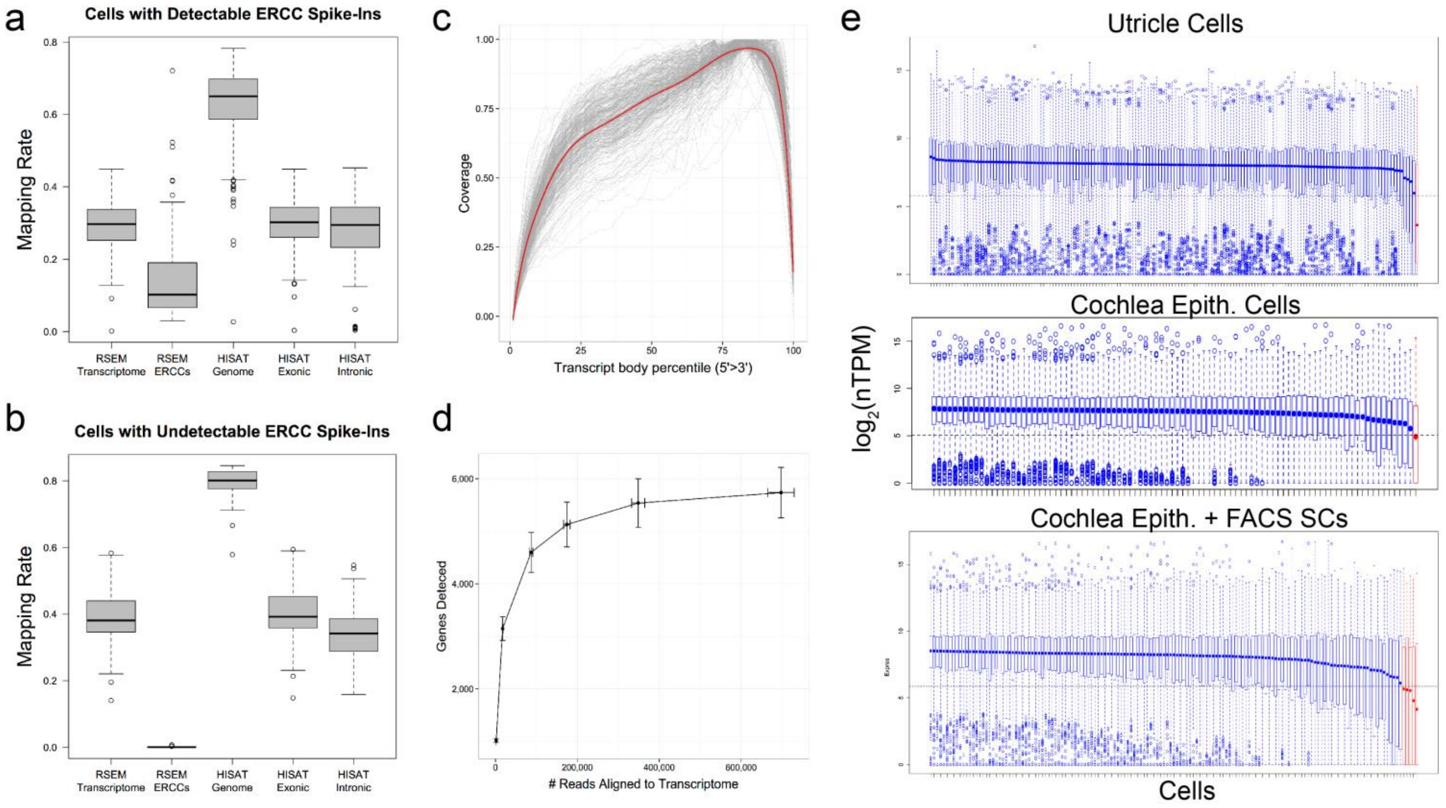

Supplementary Figure 8. Quality assessment of single-cell RNA-Seq data. a-b, Box plots of the fraction of total reads that mapped to the transcriptome, ERCC spike-ins, genome, exons, and introns. Reads mapped to transcriptome were calculated with the Bowtie/RSEM pipeline, whereas all other fractions were determined with HISAT/RNA-SeQC. The box plots represent all single cells within the dataset (including the 11 cells that were removed due to low sequencing depth or by SINGuLAR analysis). Cells were divided by IFC captures that contained detectable **(a)** or undetectable **(b)** ERCC spike-ins. Addition of ERCC spike-ins decreased the average mapping rates, but not substantially. **c,** Plot of transcript coverage for all single cells. Red line shows a polynomial fit. As reported, Smart-Seq of single cells has moderate 3' bias. **d,** Saturation plot of the average number of expressed genes detected above the LOD (TPM=1) versus average number of reads aligned to the transcriptome within seven representative cells. Cells from each of the major groups were chosen at random (one non-sensory cell, SC, and HC from the utricular and cochlear epithelial preps and one FACS-purified cochlear SC). For each cell, paired reads were down-sampled to $2x10^6$, $1x10^6$, $5x10^5$, $2.5x10^5$, $5x10^4$, and $5x10^3$ reads prior to alignment. Error bars represent s.e.m. of number of expressing genes (vertical) and number of reads aligned to transcriptome (horizontal). **e,** Results from SINGuLAR outlier analysis, presented as box plots. Each box plot represents an individual cell and shows the distribution of expression of the most stably expressed genes across all the cells (see Methods). Outlier analysis was performed separately for utricular cells, mechanically purified cochlear cells, and mechanically/FACS-purified cochlear SCs. Dashed horizontal line indicates the 15th percentile of the gene expression distribution, and cells whose median expression level falls below the threshold are red.

Supplementary Table 1. Summary of capture and sequencing statistics.

	Isolation 1	Isolation 2	Isolation 3	Isolation 4	Isolation 5	Isolation 6	Isolation 7	Isolation 8	Isolation 9
**Organ**	Utricle	Utricle	Utricle	Utricle	Cochlea	Cochlea	Cochlea	Cochlea	Cochlea
**Purification**	Mechanical	Mechanical	Mechanical	Mechanical	Mechanical	Mechanical	FACS	FACS	FACS
**# Mice**	5	4	5	5	1	1	5	6	6
**Genotype**	$Lfng^{EGFP}$; $R26R^{CAG\text{-}tdTom}$; $Gfi1^{Cre}$	$Lfng^{EGFP}$; $R26R^{CAG\text{-}tdTom}$; $Gfi1^{Cre}$	$Lfng^{EGFP}$; $R26R^{CAG\text{-}tdTom}$; $Gfi1^{Cre}$	$Lfng^{EGFP}$; $R26R^{CAG\text{-}tdTom}$; $Gfi1^{Cre}$	$Lfng^{EGFP}$; $R26R^{CAG\text{-}tdTom}$; $Gfi1^{Cre}$	$Lfng^{EGFP}$; $R26R^{CAG\text{-}tdTom}$; $Gfi1^{Cre}$	$Lfng^{EGFP}$	$Lfng^{EGFP}$	$Lfng^{EGFP}$
**# single-cell captures**	51	42	64	63	74	65	13	15	37
**# multi-cell captures**	25	46	26	29	20	25	4	1	30
**# empty captures**	20	8	6	4	2	6	79	80	39
**# GFP**	13	23	17	22	26	37	12	15	34
**# tdTomato**	17	8	39	31	7	6	NA	NA	NA
**# negative**	26	24	8	10	41	22	1	0	3
**# single cells sequenced**	37	27	45	51	50	42	12	15	34
**# outliers**	0	1	0	1	1	0	3	0	5
**# single cells analyzed**	37	26	45	50	49	42	9	14*	29
**Sequencing lane(s)**	1, 2	2	3	3, 4	5, 6, 7	6, 7	1	8, 9	9

*Note: one single cell in this capture was determined to be a HC and was not included in analysis.

Supplementary Table 2. Mouse genotyping primers.

Allele	Forward Primer	Reverse Primer
Lfng^{EGFP}	CAGTTGGCACTGGGATAGATATTACGT	GGTCGGGGTAGCGGCTGAA
Gfi1^{Cre}	GGTCGATGCAACGAGTGATGAGG	GCTAAGTGCCTTCTCTACACCTGCG
R26R$^{CAG\text{-}tdTomato}$	GGCATTAAAGCAGCGTATCC	CTGTTCCTGTACGGCATGG
R26R^{wt}	AAGGGAGCTGCAGTGGAGTA	CCGAAAATCTGTGGGAAGTC

Supplementary Table 3. Single-cell qPCR primers.

Gene ID	Target RefSeq ID	Forward Primer	Reverse Primer
Actb	NM_007393.3	CCCTAAGGCCAACCGTGAAA	AGCCTGGATGGCTACGTACA
Gapdh	NM_008084.2	AGACGGCCGCATCTTCTT	TTCACACCGACCTTCACCAT
Cdh2	NM_007664.4	TCCAGAGGACCCTTTCCTCA	GTGACGCTGTATCTCAGGGAA
Lfng	NM_008494.3	TCGATCTGCTGTTCGAGACC	CCTCCCCATCAGTGAAGATGAA
Heyl	NM_013905.3	GTCCCCACTGCCTTTGAGAA	TCCACGGTCATCTGCAAGAC
Sox2	NM_011443.3	CCTGCAGTACAACTCCATGAC	TGCGAGTAGGACATGCTGTA
Isl1	NM_021459.4	GGACAAGAAACGCAGCATCA	GTTCCTGTCATCCCCTGGATA
Jag1	NM_013822.4	TCCCAAGCATGGGTCTTGTA	GATGCACTTGTCGCAGTACA
Gli3	NM_008130.2	CCGTAGCAGCTCTTCAGCAA	GGGTAGGTGAAGCTCAATGCA
Notch3	NM_008716.2	CCATGCCGATGTCAATGCA	TAGCCTCCACGTTGTTCACA
Notch2	NM_010928.2	TGGTTCTGGGACAAGTGAACA	ACAGCAAAGCCTCATCCTCA
Maml2	NM_001013813.3	AGACCAACCATGGAGCAGAA	GTTCATCTGATCCTGAGGGGAA
Hes1	NM_008235.2	TGAAGCACCTCCGGAACC	CGCGGTATTTCCCCAACAC
Hey1	NM_010423.2	CGAGACCATCGAGGTGGAAA	ATGTCGTTGGGGACATGGAA
Slc1a3	NM_148938.3	AATGCCTTCGTTCTGCTCAC	TTATACGGTCGGAGGGCAAA
Egr3	NM_018781.2	CGACTCGGTAGCCCATTACAA	GTCAGACCGATGTCCATCACA
Notch1	NM_008714.3	GGACGGCGTGAATACCTACA	GACATTCGTCCACATCCTCTGTA
Sox9	NM_011448.4	AGTACCCGCATCTGCACAA	GTCTCTTCTCGCTCTCGTTCA
Cdh1	NM_009864.2	ATTGCAAGTTCCTGCCATCC	CAGTAGGAGCAGCAGGATCA
Myo6	NM_001039546.2	TTTTGAGGAAGCCGGAAGCA	AGCAGCTCAGCACAGTATTCC
Pou4f3	NM_138945.2	ATGCGCCGAGTTTGTCTCC	GCCAGCAGGCTCTCATCAAA
Dll1	NM_007865.3	TGGCTGGAAAGGCCAGTAC	CCCTGGTTTGTCACAGTATCCA
Atoh1	NM_007500.4	CCGTCCTTCAACAACGACAA	TCCGACAGAGCGTTGATGTA
Pax2	NM_011037.3	CCATGGCTGTGTCAGCAAAA	GCTTGGAGCCACCAATCAC
Dach1	NM_007826.2	TGACATGGGGCATGAGTCAAA	TCTTGCGGTTGGTGTGGAA
Jag2	NM_010588.2	CTCGTCGTCATTCCCTTTCA	GGTGTCATTGTCCCAGTCC
Gfi1	NM_010278.2	TGAGCCTGGAGCAACACA	AGCGTGGATGACCTCTTGAA
Slc17a8	NM_182959.3	ACCACAACCGCTGTCAGAAA	AAATCCAACCACCAGGAGCAA
Barhl1	NM_001164186.1	AATACCTGAGCGTGCAAGAC	CCGTCTGTCGCTTCCATTTA
Pvalb	NM_013645.3	TGCCAGAGACTTGTCTGCTA	CAGCCACCAGAGTGGAGAA
